# Predicted Spatial Spread of Canine Rabies in Australia

**DOI:** 10.1371/journal.pntd.0005312

**Published:** 2017-01-23

**Authors:** Simon P. Johnstone-Robertson, Peter J. S. Fleming, Michael P. Ward, Stephen A. Davis

**Affiliations:** 1 RMIT School of Science, Mathematical and Geospatial Sciences, RMIT University, Melbourne, Victoria, Australia; 2 School of Environmental and Rural Science, University of New England, Armidale, NSW, Australia; 3 Vertebrate Pest Research Unit, Orange, NSW, Australia; 4 Sydney School of Veterinary Science, University of Sydney, Narellan, NSW, Australia; Wistar Institute, UNITED STATES

## Abstract

Modelling disease dynamics is most useful when data are limited. We present a spatial transmission model for the spread of canine rabies in the currently rabies-free wild dog population of Australia. The introduction of a sub-clinically infected dog from Indonesia is a distinct possibility, as is the spillover infection of wild dogs. Ranges for parameters were estimated from the literature and expert opinion, or set to span an order of magnitude. Rabies was judged to have spread spatially if a new infectious case appeared 120 km from the index case. We found 21% of initial value settings resulted in canine rabies spreading 120km, and on doing so at a median speed of 67 km/year. Parameters governing dog movements and behaviour, around which there is a paucity of knowledge, explained most of the variance in model outcomes. Dog density, especially when interactions with other parameters were included, explained some of the variance in whether rabies spread 120km, but dog demography (mean lifespan and mean replacement period) had minimal impact. These results provide a clear research direction if Australia is to improve its preparedness for rabies.

## Author Summary

Canine rabies typically persists in developing countries where stray and unvaccinated, free-roaming domestic dogs account for a substantial proportion of the population. In this paper we investigate whether sustained canine rabies transmission can occur within the wild dog population of Australia, which comprises dingoes and their hybrids with domestic dogs. To do this we simulated the transmission of rabies between wild dogs across a sizeable landscape area. Whenever the disease spread 120 km we considered an epidemic as having occurred and recorded the length of time it took to do so. This allowed us to estimate the probability the sustained transmission of canine rabies will occur (21%) upon its introduction along with the speed at which it will spread (67 km/year). We also determined that wild dog movements and behaviour (for which there is the least amount of information available at present) together with wild dog density are the most important factors influencing these two outcomes, whereas wild dog demography (including average lifespan and average replacement period) had minimal impact. Future research should therefore focus on studying wild dog movements and behaviour to improve Australia’s readiness to control and prevent canine rabies transmission and establishment.

## Introduction

Rabies is a zoonotic disease caused by a virus of the genus *Lyssavirus*. Annually, it causes an estimated 61,000 human deaths in over 150 countries and territories where it is endemic [1–3]. The canine strain of the virus is the most widely distributed globally [4], with dogs accounting for the vast majority (up to 99%) of human rabies infections and deaths [3, 5].

A key feature of rabies that assists in its spread and persistence is the potentially long incubation period [6, 7]. For canine rabies the incubation period can vary from 10 days to 6 months, but for most cases lasts between 2 weeks and 3 months [2]. The extended incubation period often allows rabies to remain undetected and enter new areas [8, 9], and is one reason why the eventual incursion of canine rabies into Australia is likely [2, 10].

Australia is historically free of canine rabies, with only the bat strain (Australian bat lyssavirus) endemic on the continent [1, 2, 10, 11]. In South-East Asia, though, canine rabies is endemic and currently spreading eastward along the Indonesian archipelago such that it is now less than 300 km from the northern Australian border [2, 8–12]. Two locations have been identified as probable entry points, namely Arnhem Land in the Northern Territory and Cape York Peninsula in Queensland [2]. Canine rabies introduction is anticipated to occur via a sub-clinically infected dog illegally brought into the country by means of a fishing vessel, pleasure craft, or boat continuing cross-cultural traditions established centuries ago [13, 14].

Incursion alone, however, is insufficient for disease establishment. Instead, the index case will also have to contact, bite, and successfully transmit the virus to at least one other resident dog. Several factors make this first transmission event possible: (1) although much of northern Australia is largely uninhabited, many of the remote communities are on or close to the coast, (2) free-roaming dogs are common in these communities [15, 16], and (3) wild dogs, comprising mostly dingoes and their hybrids with free-roaming domestic dogs, are ubiquitous across northern Australia [17, 18].

Sparkes et al. [19] have noted Australia stands much to lose should canine rabies become established in its wild dog population. The first, and most significant, loss could be that of human life, especially in peri-urban areas where wild and domestic dogs frequently coincide and the likelihood of human exposure is greatest. There will also be economic losses due to reduction in ecotourism, rabies-related livestock losses, and the large-scale costs associated with mass vaccination of domestic dogs and wildlife, and the administration of post-exposure prophylaxis to humans. Perhaps harder to quantify, but nevertheless important, is the impact rabies would have on the Australian nation’s affinity for, interaction with, and conservation of wildlife. Australia is renowned for, and prides itself on, its unique fauna with which people frequently interact. This forms a key component to the national image as a whole. The presence of rabies in wild dogs, however, would certainly lead to greater restraint on wildlife interactions due to the fear of potential rabies exposure. This will be especially true should other native wildlife besides wild dogs also be at risk of canine rabies infection. Consequent to each of these foreseeable losses, risk assessment for the sustained transmission of canine rabies within the Australian wild dog population has been identified as a national research priority [10].

Mathematical models of infectious disease transmission are playing an increasing role in informing and directing public health policy, as well as providing opportunities for authorities to explore control options [20]. An example is canine rabies in Australia, for which there is an absence of epidemiological data. Spatial rabies transmission in small (≤2.2 km^2^), remote communities of northern Australia with high densities of domestic dogs (≥137 dogs/km^2^) has been modelled [21] but there are no existing models for wild dogs which are territorial by nature and occupy an expansive Australian landscape at much lower densities. We therefore present a stochastic transmission network (percolation) model for the spatial spread of canine rabies through the Australian wild dog population that allows us to estimate the probability a canine rabies epidemic will occur, given its introduction, and the geographic rate at which rabies will spread. We also conduct a global sensitivity analysis to identify where knowledge is most critical for predicting model outcomes.

## Methods

### Wild dog contact networks

**Fig 1:**
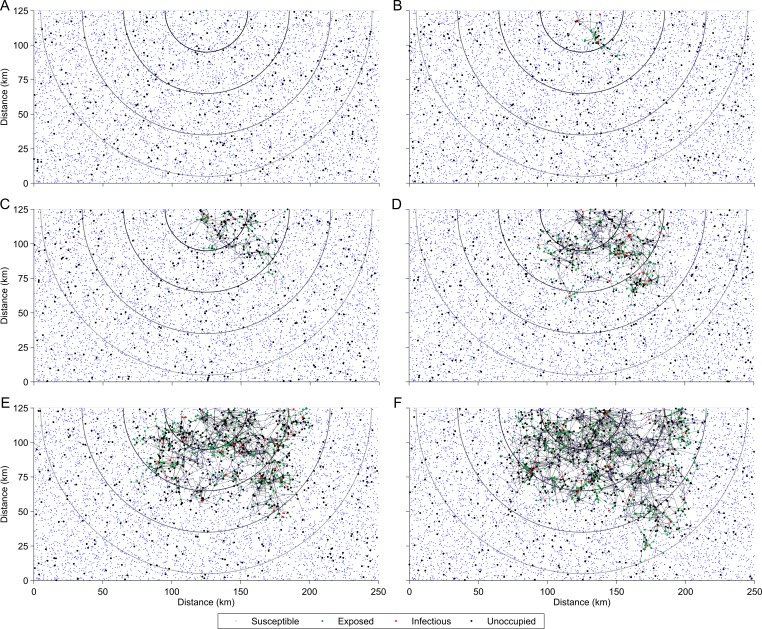
A simulated canine rabies epidemic in the Australian wild dog population. The spread of canine rabies (A) 0, (B) 24, (C) 46, (D) 70, (E) 92, and (F) 116 weeks after the introduction of a single sub-clinically infected (exposed) index case into the Australian wild dog population. Each node, representing a potential wild dog home range centroid, is either occupied by a (blue) susceptible, (bold green) exposed, or (bold red) infectious dog, or alternatively is (bold black) unoccupied. An occupied node’s location corresponds to the average position of the dog that occupies the home range. An edge between two nodes represents a transmission event which occurred sometime in the past between two dogs (one infectious, one susceptible) that either currently occupy or previously occupied the nodes (the infection (occupancy) status of each dog (node) may have changed in the time since the transmission event). Edges are shown for all transmission events since the start of the epidemic (not only recent transmission) such that a node may be adjacent to multiple edges by the time rabies percolates (116 weeks for this particular transmission network realization) beyond the fourth milestone distance (lightest grey semi-circle, 120 km). The parameter values used to generate this transmission network realization were: landscape width = 250 km and height = 125 km, wild dog density = 0.20 dogs/km^2^, wild dog sociability shape parameter = 30 and scale parameter = $\left(\frac{1}{30}\right)\sqrt{2}$ (gamma distribution), probability of a bite given contact = 0.50, transmission probability given a bite = 0.50, spatial scale parameter *λ* = 0.4 km^-1^, mean infectious period = 3 days (exponential distribution), model time step *δ* = 1 day, incubation period shape parameter = 7 and scale parameter = 4 (gamma distribution), mean wild dog lifespan = 3 years (exponential distribution), and mean replacement period = 50 days (exponential distribution).

We simulated the spread of canine rabies through the Australian wild dog population by implementing the model of Davis [22]. Each simulation began by distributing nodes (representing the centroids of potential wild dog home ranges) uniformly at random across a landscape of width 250 km and height 125 km (Fig 1). The density of nodes distributed in each simulation was calculated using the formula derived in S1 Appendix.

Because the incubation period of rabies is on the order of weeks to months, and because wild dogs inhabit an expansive Australian landscape, we expected the spread of rabies through the wild dog population to take on the order of months to years. Consequently, we included wild dog demographic processes in the model whereby home ranges become vacant through natural mortality and are subsequently reoccupied through migration or recruitment. Each node was therefore either occupied by a wild dog or unoccupied at any given point in time during a simulation.

**Fig 2:**
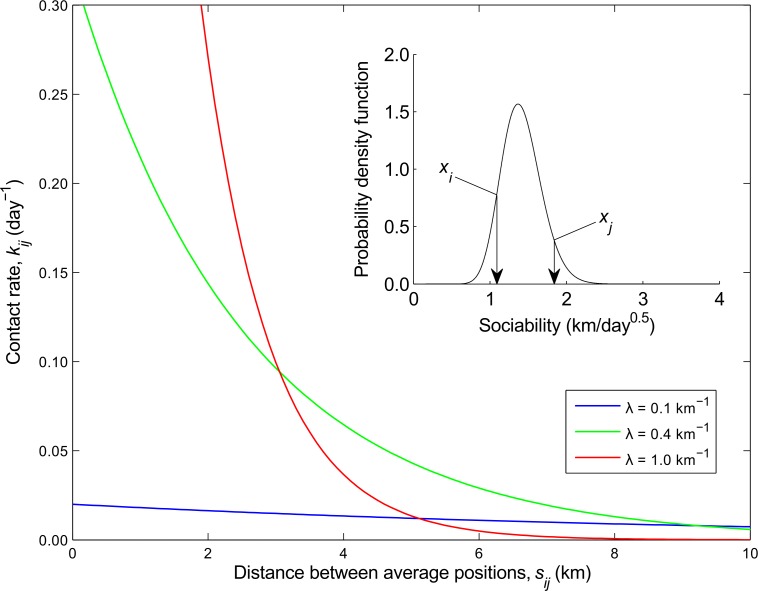
The proposed wild dog contact rate as a function of geographic distance. The contact rate, *k*_*ij*_, of two wild dogs *i* and *j*, with sociabilities *x*_*i*_ = 1.09 km/day^0.5^ and *x*_*j*_ = 1.84 km/day^0.5^, as a function of the distance between their average positions, *s*_*ij*_, for three values of the spatial scale parameter, *λ*: (blue) 0.1 km^-1^, (green) 0.4 km^-1^, and (red) 1.0 km^-1^. The inverse of the contact rate is equal to the mean time between contact events such that a contact rate of *k*_*ij*_ = 0.2 day^-1^ implies dogs *i* and *j* come into contact on average once every 5 days. (Inset) The wild dog sociability distribution (gamma distribution: shape parameter = 30, scale parameter = $\left(\frac{1}{30}\right)\sqrt{2}$) from which *x*_*i*_ and *x*_*j*_ were sampled and which was also used to generate the transmission network realization in Fig 1. The mean value of this distribution, given by the product of the shape and scale parameters, equates to wild dogs traversing 2 km^2^/day on average.

When a node was occupied, its location represented the average position of the dog inhabiting the corresponding home range. Consequently, one should not think of the dogs as being fixed at these locations. Instead, each dog makes contact with other dogs over time by way of their normal everyday movement across the landscape for activities such as foraging, finding water, taking shelter, communicating, finding a mate, and raising young [2, 23, 24]. We propose the rate at which two dogs *i* and *j* contact one another, *k*_*ij*_, is a function of the Euclidian distance, *s*_*ij*_, between the two dogs’ mean positions (nodes), as well as their individual inclination towards making contact with other dogs (sociability), *x*_*i*_ and *x*_*j*_ respectively. Specifically, we defined the contact rate as

$$k_{ij}=\lambda^{2}e^{-\lambda s_{ij}}x_{i}x_{j},$$ (1)

where *λ* (units: km^-1^) is a spatial parameter that captures the scale over which distance between two dogs affects their rate of contact. For large values of *λ* the maximum contact rate (*s*_*ij*_ = 0) is high and the contact rate declines sharply with distance (Fig 2) such that dogs effectively only come into contact with their nearest neighbours. Conversely, for small values of *λ* the maximum contact rate is low but dogs come into contact with other dogs that are far away almost as often as their nearest neighbours. Eq 1 has the property that the average number of contacts a dog has with other dogs over some period of time is independent of *λ* [22]. Another appealing feature, revealed by dimensional analysis (S1 Appendix), is that a dog’s sociability, *x*_*i*_, is related to the area of land it traverses per day.

### Canine rabies transmission networks

To simulate the spread of rabies we employed long-range percolation [22, 25, 26] on the dynamic wild dog contact networks described above. Rabies was introduced into the wild dog population by selecting the dog closest to the centre of the upper boundary of the landscape as the index case and infecting it. Thereafter a directed edge from an infectious dog *i* to a susceptible dog *j*, representing a successful transmission event, was generated with probability

$$p_{ij}=1-e^{-\tau k_{ij}\delta },$$ (2)

where *τ* is the transmission probability given contact and *δ* = 1 day is the model time step. Dogs exposed to infection were assigned a gamma distributed incubation period after which they were rabid (infectious) for an exponentially distributed period of time [27–29]. When a dog died (either due to natural mortality or disease) its corresponding node was rendered ‘unoccupied’ and remained so until a new susceptible dog (with its own unique sociability) arrived. We refer to the average length of time until an unoccupied home range was reoccupied as the ‘replacement period.’

In each simulation rabies was transmitted from dog to dog and eventually either died out or progressed 120 km from the index case (lightest grey semi-circle, Fig 1). Because, in spatial disease systems, the percolation of disease beyond various milestone distances can be construed as alternative definitions of an epidemic [22, 25], we considered four arbitrarily chosen milestone distances, namely 30, 60, 90, and 120 km (dark to light grey semicircles, Fig 1).

### Global sensitivity analysis

For each transmission network realization (simulation) we recorded 11 outcome variables of interest:

- (1–4) the binary outcomes indicating whether or not the disease percolated beyond each of the four milestone distances,
- (5) the number of dogs infected by the index case during its infectious period,
- (6–9) the time taken for the disease to percolate beyond each milestone distance given the disease percolated beyond each respective milestone distance,
- (10) the total number of nodes within the first milestone distance, and
- (11) the number of dogs (occupied nodes) within the first milestone distance before rabies was introduced.

If the disease percolated beyond the fourth milestone distance (120 km) a further 3 conditional outcomes were recorded at the time of percolation:

- (12) the reduction in wild dog density within the first milestone distance,
- (13) the proportion of infected (exposed), but not yet infectious, dogs within the first milestone distance, and
- (14) the proportion of infectious dogs within the first milestone distance.

To quantify the sensitivity of outcomes 1–5 to each model input variable we performed a global sensitivity analysis. In particular, we calculated Sobol’s (sensitivity) indices because they describe the proportional contribution of each input variable’s uncertainty to the variance of each respective outcome [30, 31]. In addition, Sobol’s indices capture any interaction effects between the input variables and do not require or assume any particular model structure (e.g. linear or monotonic) [30].

To calculate Sobol’s indices we implemented the Monte Carlo procedure proposed by Saltelli [31] for which the technical details are described in S1 Appendix. Briefly, though, we defined a distribution for each input variable by specifying a range of values (see Parameterization section below) that captures either its natural variation (e.g. geographic, seasonal, etc.) or any other uncertainty in its value (e.g. no published estimates) and assuming a uniform distribution across this range. Next, each input variable distribution was sampled 50,000 times (assuming independent input variables) by employing (quasi-Monte Carlo) Sobol sequences, which are deterministic, uniformly distributed sequences that ensure more uniform sampling over the input parameter space than is achieved by traditional pseudo-random sampling. Doing so facilitates faster convergence of the sensitivity indices such that fewer samples are required [32]. The sample values were then stored in two matrices **M** and **M**′ (half in each matrix) such that each row of the matrices denoted a sample vector from the model input parameter space and each column contained the sample values of a single input variable. For each input variable, e.g. *y*_*j*_, a further two matrices **N**_*j*_ and **N**_¬*j*_ were defined as a combination of the columns of **M** and **M**′ (see S1 Appendix). Thus for our model with 6 input variables (5 biological, 1 random number generator seed–see below) a total of 14 matrices of size (25,000 × 6) were generated. Next, a transmission network realization was generated for each row (sample vector) of each matrix. This resulted in 14 different, but closely related, distributions of values for each model outcome from which the sensitivity indices were subsequently calculated using the formulas described in S1 Appendix (Equations S17—S20).

Two sensitivity indices were calculated for each input and outcome variable pair, namely the first-order and total effects. The first-order effect, *S*_*j*_, is the average proportional reduction in the variance of an outcome variable, *z*, when an input variable, *y*_*j*_, has no uncertainty (i.e. it is assigned a fixed value). The greater the value of *S*_*j*_ the more influential is input variable *y*_*j*_ in determining the value of *z*. Conversely, the total effect, $S_{j}^{T}$, is defined as the average proportion of variance that remains when all input variables are assigned fixed values (i.e. are known) except for input variable *y*_*j*_. The total effect may be interpreted as the sum of an input variable’s first-order effect and any additional effects resulting from its interaction with other input variables.

We note here that because the canine rabies model is a stochastic simulation model, even if all biological input variables were assigned fixed values, model outcomes would still vary from one simulation to the next. That is to say, model outcome variance is not completely explained by biological input variable uncertainty alone. This is important because this additional variance will affect the accuracy of global sensitivity indices estimated using the procedure above. To address this, the random number generator seed (which explains all model variance over and above that accounted for by the biological input variables) must be treated as an additional input variable, sampled from its own unique distribution, and assigned a value at the start of each simulation (as is done for the biological input variables). For the rabies model, we sampled the seed from a discrete uniform distribution with range [0,2^32^−1]. This range was selected as it includes all permissible integer values for the random number generator seed (MATLAB R2012b), thereby ensuring no bias was inadvertently introduced into model outcome distributions by falsely limiting the range of values the seed could assume (this is especially important when there are strong interactions between a model’s input variables). An important indication that sampling the random number generator seed has been done correctly is that the seed accounts for a non-negligible proportion of model outcome variance (at the very least this should be true for the total effect) and that when model outcomes are plotted against the seed value a horizontal trend line is observed (i.e. model outcomes should be independent of the seed).

Lastly, to estimate 95% confidence intervals for Sobol’s indices we employed the bootstrapping procedure prescribed in [33].

### Parameterization

Because many wild dogs are hybrids of dingoes and modern domestic dog breeds [34] we assumed canine rabies pathogenesis parameters for wild dogs will be similar in value to those reported in the literature for domestic dogs. More information is available for canine rabies pathogenesis parameters than those relating to wild dog ecology in northern Australia [2, 10, 27, 28, 35, 36]. Consequently, we assigned disease related parameters point estimates obtained from the literature whereas for ecological parameters we considered a range of feasible values guided by a combination of expert opinion and the literature (Table 1). If absolutely no information was available for a particular ecological parameter, a range of values spanning an order of magnitude was selected based on mechanistic reasoning.

**Table 1:** Canine rabies model parameter point estimates and input variable distributions.

Parameter (Units)	Point Estimate/Distribution	Literature
Model time step, δ (days)	1	
Random number generator seed^a^^,^^b^	*U*{0,2^32^ − 1}	
**Canine rabies pathogenesis parameters**		
Incubation period shape parameter	7	[27, 28, 35, 37]
Incubation period scale parameter	4	[27, 28, 35, 37]
Mean infectious period (days)	3	[27, 36, 37]
Probability a rabid dog bites another dog given contact	0.5	Assumed
Transmission probability given a bite	0.5	[27]
**Wild dog ecological parameters**		
Population density^a^ (dogs/km^2^)	*U*(0.05,0.38)	[2, 38]
Wild dog sociability shape parameter	30	Assumed
Wild dog sociability scale parameter^a^	$U\left(\frac{1}{30}\sqrt{0.5},\frac{1}{30}\sqrt{5}\right)$	Assumed
Spatial scale parameter^a^, λ (km^-1^)	*U*(0.1,1.0)	Assumed
Mean lifespan^a^ (years)	*U*(2,4)	[10, 23, 24]
Mean replacement period^a^ (days)	*U*(1,100)	[38]

^a^For each of the six input variables 50,000 values were sampled from their respective uniform distributions.

^b^Values for the random number generator seed were sampled from the discrete uniform distribution.

At present, there are three published, field-data derived estimates for the mean incubation period of canine rabies. Hampson et al. [28] and Hampson et al. [27] report values of 25.5 days and 22.3 (95%CI: 20.0–25.0) days respectively, derived directly from Tanzanian rabid dog natural infection and contact tracing data. Coleman et al. [37], on the other hand, obtain their estimate of 4.18 (SE: 0.27) weeks from a thesis [35] which calculates the mean incubation period from observed canine rabies cases in Zimbabwe. We therefore assumed the incubation period was gamma distributed (as observed by [27]) with a mean of 28 days (fixed shape parameter = 7, fixed scale parameter = 4). Under these assumptions, 93.5% of simulated incubation periods in the transmission network model lasted between 2 weeks and 3 months, with the remaining 6.5% shorter than 2 weeks.

The infectious period of canine rabies is typically much shorter than the incubation period. Tepsumethanon et al. [36] reported that naturally infected rabid dogs in Thailand survived a median of 4 (95%CI: 3.7–4.3) days after first displaying symptoms (abnormal behaviour or bite event). Two field-data derived mean infectious periods of 3.1 (95%CI: 2.9–3.4) days and 0.81 (range: 0.29–1.71) weeks have also been published by Hampson et al. [27] and Coleman et al. [37] respectively. There is some evidence supporting a gamma distributed infectious period [27]. Nevertheless, the infectious period is often modelled using the closely related exponential distribution [28, 29]. We adopted this same approach here assuming a fixed mean of 3 days such that 96.4% of simulated infectious periods were shorter than 10 days.

In Eq 2, *τ* is defined as the transmission probability given contact. Rabies transmission, however, requires that an infectious dog inoculate a susceptible dog by biting it rather than by merely being in its vicinity. Thus, assuming independence, *τ* is equivalent to the probability a rabid dog bites another dog given contact (for which we assumed a fixed value of 0.5) multiplied by the probability of successful transmission given a rabid dog bites a susceptible dog (estimated by Hampson et al. [27] to be 0.49 (95%CI: 0.45–0.52) and which we took as 0.5).

No estimates for the population density of wild dogs (occupied nodes) in tropical northern Australia, where a rabid dog incursion is most likely, have been published to date. Therefore, to select a range of feasible values we considered wild dog population density estimates obtained for other regions around Australia (which have different terrain types, climates, and levels of anthropogenic impact). In general, wild dog density appears to depend largely on landscape carrying capacity, with lower densities observed in arid regions (0.08 dogs/km^2^) and higher densities in higher-rainfall areas (0.14–0.3 dogs/km^2^) [2]. It is also likely that dog densities have responded positively to agriculture since anthropogenic resources subsidize both food (sheep, cattle, goats, kangaroos, and rabbits) and water (nowhere in the cattle zone is further than 10 km from water) [38, 39]. Conversely, wild dog densities may be reduced by human-related control measures such as baiting. To account for the variation in wild dog densities resulting from each of these factors we considered densities within the range 0.05–0.38 dogs/km^2^.

The literature provides no estimates for the area of land a wild dog traverses per day, reporting only home ranges, which are not rates and therefore not equivalent [2, 10]. Nevertheless, because wild dog home ranges vary between 10 and 100 km^2^ [10], and because a wild dog probably only covers a small portion of its home range each day (although potentially frequenting some areas more than once each day), we considered mean values for the area of land traversed per day from a plausible range spanning an order of magnitude (0.5–5 km^2^/day). Assuming a wild dog’s sociability was equal to the square root of the area it traverses per day (S1 Appendix) we then calculated a range for the mean sociability of $\sqrt{0.5}–\sqrt{5}$ km/day^0.5^. To assign each wild dog a unique sociability, and thereby incorporate individual contact heterogeneity into the model, we assumed wild dog sociabilities were gamma distributed. Specifically, we assumed the sociability shape parameter takes a fixed value (arbitrarily chosen to be 30) whilst the sociability scale parameter was sampled from the range $\left[\frac{1}{30}\sqrt{0.5},\frac{1}{30}\sqrt{5}\right]$ to ensure the desired range of values for the mean area of land traversed per day.

There are no recorded estimates in the literature for the introduced spatial scale parameter *λ*. Nor are there any appropriate data (e.g. proximity log or GIS tracking data) relating the contact rate of wild dogs to the distance between their mean positions that can be used to estimate *λ*. Wild dog contact distance distributions, however, are likely to be highly left-skewed [38]. From a mechanistic perspective, it seems reasonable to expect wild dog contact rates to decline substantially over distances between 1 km and 10 km. We therefore sampled *λ* from a range of values spanning an order of magnitude (0.1–1.0 km^-1^), where for *λ* = 0.1 km^-1^ the wild dog contact rate declines by 50% every 7 km and for *λ* = 1.0 km^-1^ it declines by 50% every 0.7 km.

The natural mortality of wild dogs is a complex parameter that is most likely age- (highest in juveniles <18 months) and density-dependent; the precise relationship, however, has not yet been quantified. It has been noted, though, that wild dogs live up to 10 years, with most dying by 5–7 years [10, 23, 24]. Therefore, to maintain model simplicity we assumed wild dog lifespan was exponentially distributed and sampled the mean value from a range of 2–4 years such that the probability a wild dog lived ≥10 years varied from 0.7–8.3%.

To maintain model simplicity we assumed the replacement period was exponentially distributed and sampled the mean value from a range of 1–100 days based on expert opinion [38].

## Results

### Probability of a canine rabies epidemic

Of the 50,000 transmission network realizations obtained from evaluating the model on the rows of matrices **M** and **M**′, canine rabies percolated 30, 60, 90, and 120 km a total of 11,306 (23%), 10,782 (22%), 10,695 (21%), and 10,672 (21%) times respectively. We therefore estimate there is a 21% probability the introduction of a single sub-clinically infected dog into the wild dog population of Australia and subsequent transmission of rabies virus will result in a canine rabies epidemic. Furthermore, because there is little difference between the probabilities rabies percolates 30 km and 120 km, once an epidemic starts it is unlikely to stop naturally. We also note that for 99.4% of the simulations in which rabies percolated 120 km, rabies was still present within milestone 1 (30 km) at the time of percolation beyond milestone 4. This suggests the percolation of rabies 120 km or further is a reasonable indicator that canine rabies has become established in the wild dog population (at least in the region close to where the initial transmission event occurred).

### Basic reproduction number

The basic reproduction number (*R*_0_) was estimated as the mean number (1.09, 95%CI: 1.07–1.10) of wild dogs infected by the index case over all 50,000 transmission network realizations.

### Rate of spread

**Fig 3:**
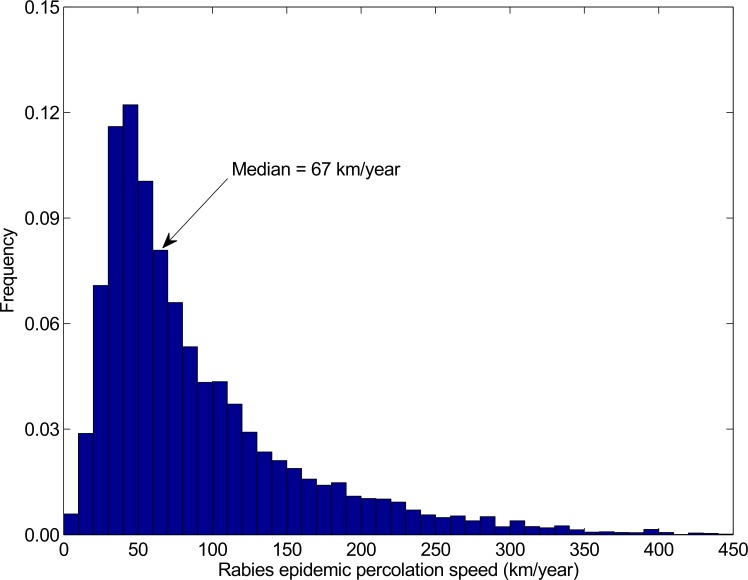
The distribution of canine rabies percolation speeds. The distribution of speeds canine rabies might spread through the Australian wild dog population, estimated from the time taken for the disease to percolate beyond the fourth milestone distance (120 km) given that it percolates beyond the fourth milestone distance.

Given the current absence of epidemiological and ecological data for canine rabies in Australian wild dogs we used the time taken for rabies to percolate beyond the fourth milestone distance (120km), given that it percolated 120km, to plot a baseline distribution of speeds the disease might spread across the Australian landscape (Fig 3). The distribution generated indicates a mean (median) rate of spread of 90 (67) km/year.

### Relationship between outcome and input variables

**Fig 4:**
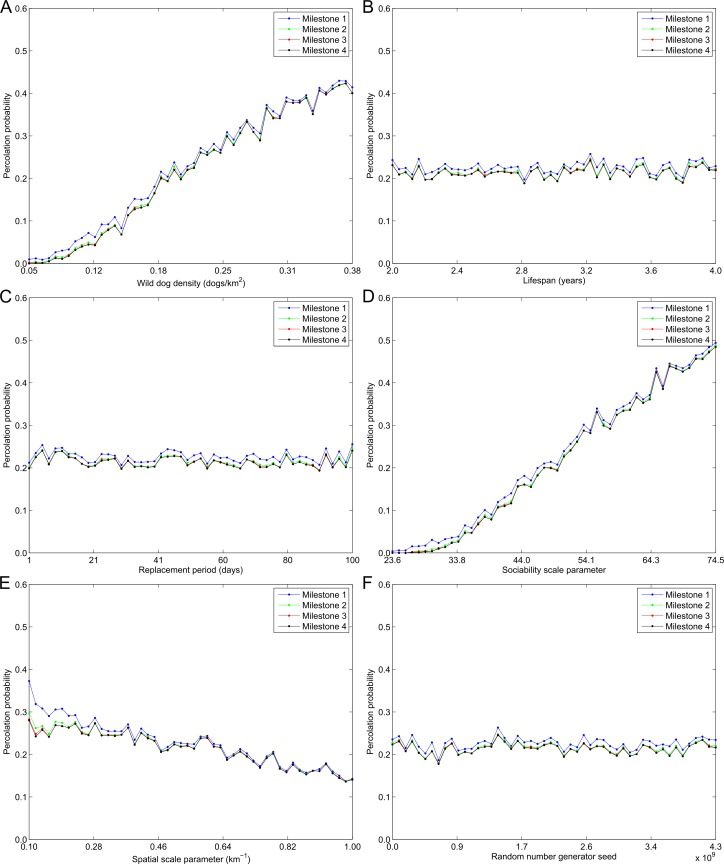
The percolation probability versus model input parameters. The probability canine rabies percolates (blue, milestone 1) 30 km, (green, milestone 2) 60 km, (red, milestone 3) 90 km, and (black, milestone 4) 120 km as a function of (A) wild dog density, (B) mean lifespan, (C) mean replacement period, (D) sociability scale parameter, (E) spatial scale parameter, *λ*, and (F) random number generator seed.

**Fig 5:**
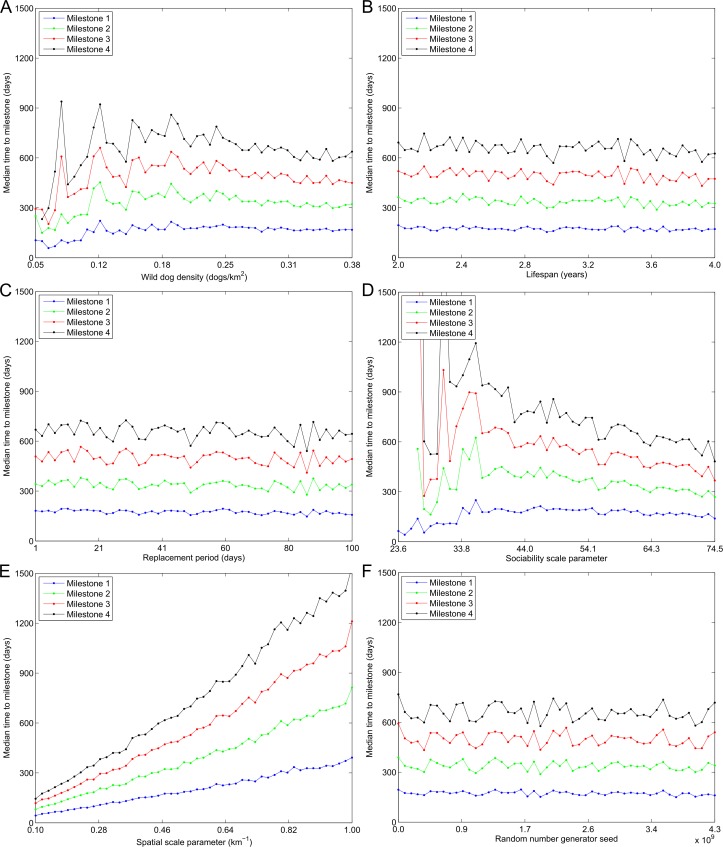
The time to milestone versus model input parameters. The median time taken for canine rabies to percolate (blue, milestone 1) 30 km, (green, milestone 2) 60 km, (red, milestone 3) 90 km, and (black, milestone 4) 120 km as a function of (A) wild dog density, (B) mean lifespan, (C) mean replacement period, (D) sociability scale parameter, (E) spatial scale parameter, *λ*, and (F) random number generator seed, given rabies percolates beyond each respective milestone distance.

The distributions of model outcomes for the 50,000 transmission network realizations were used to investigate the relationships between the outcomes and six input variables (Figs 4 and 5, S1 Fig–S6 Fig). To do this, each simulation was binned by input variable value and thereafter the median (or mean) value of each outcome in each bin calculated. Here we report the results for the percolation probability and time to percolation. We refer the reader to S1 Fig–S6 Fig for the results of the remaining model outcomes.

In Fig 4 the probability canine rabies percolates beyond each milestone distance is plotted as a function of each input variable. Once again we observe from the closely overlapping curves that there is little difference between the probability rabies will spread 30 km (milestone 1) or 120 km (milestone 4). Also noticeable is that as values for wild dog density or the sociability scale parameter increase the probability of percolation increases rapidly. Specifically, for wild dog densities below 0.15 dogs/km^2^ and a sociability scale parameter less than 36.0 the probability rabies will percolate beyond milestone 4 is less than 12% and 5% respectively. Once wild dog densities rise above 0.35 dogs/km^2^ and the sociability scale parameter increases to 67.0 the probability of percolation exceeds 40%. Lastly, the more territorial wild dogs are in their behaviour (i.e. as values for the spatial scale parameter, *λ*, get larger) the lower the probability of percolation.

In Fig 5 the median time for rabies to percolate beyond each milestone distance, given rabies percolated beyond each respective milestone distance, is shown as a function of each input variable. Three features are immediately apparent, the first of which is that the curves are equidistant. This indicates that the wave front speed was constant over time and with distance from the index case. The second feature is that the median time for percolation grows almost linearly as the spatial scale parameter, *λ*, increases in value. Thirdly, we note that at low values for wild dog density and the sociability scale parameter the median time to percolation is highly variable. This is explained by the fact that in this region of parameter space canine rabies very seldom percolated beyond the milestone distances (see Fig 4). Consequently, only a small number of simulations contribute to the calculation of the median time to percolation in each bin resulting in greater variance between bins. Once wild dog density and the sociability scale parameter are larger than 0.15 dogs/km^2^ and 40.0 respectively, such that the probability of percolation is greater than 0.10, the estimates for the median time to percolation become less variable.

### Global sensitivity analysis

**Fig 6:**
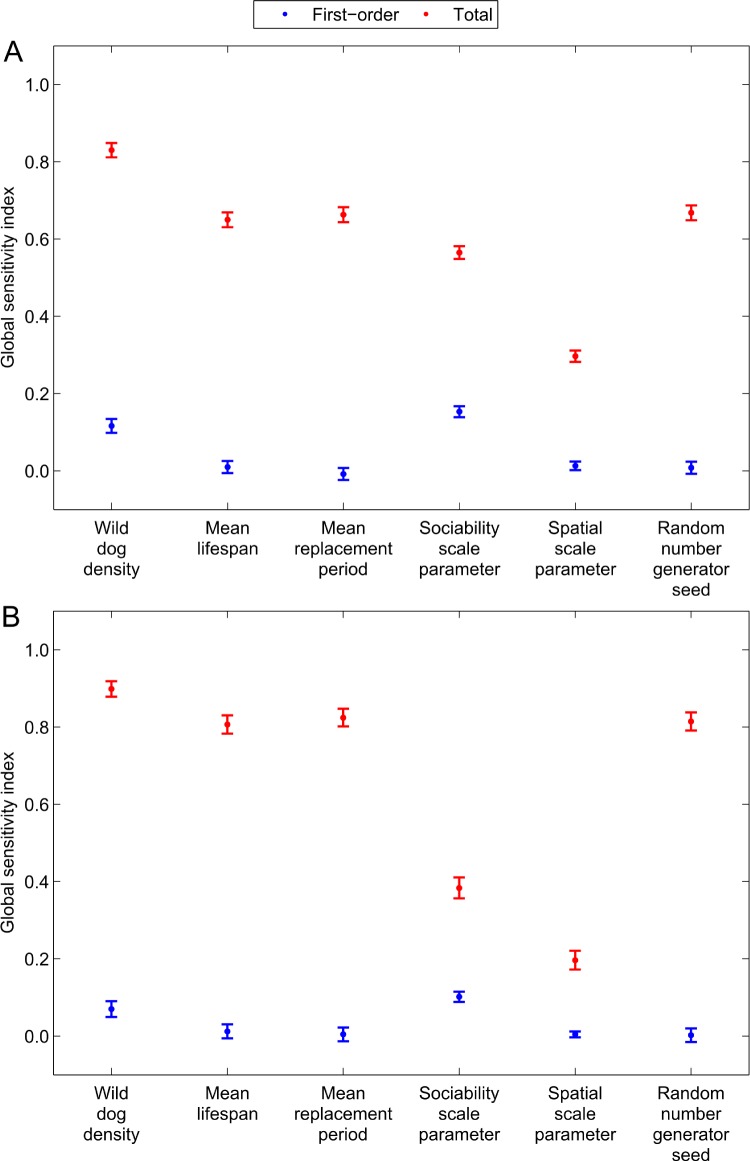
Global sensitivity indices and bootstrap-estimated 95% confidence intervals. The (blue) first-order and (red) total effect global sensitivity indices with bootstrap-estimated 95% confidence intervals for the six model input variables (wild dog density, mean lifespan, mean replacement period, sociability scale parameter, spatial scale parameter, and random number generator seed) and two model outcomes: (A) the binary outcome indicating whether or not rabies percolates beyond the fourth milestone distance (120 km) and (B) the number of dogs infected by the index case during its infectious period.

The results of the global sensitivity analysis are reported in Tables 2 and 3, and plotted in Fig 6. Before describing them in detail, it is instructive to point out that some of the sensitivity indices are negative even though by definition they should fall within the range [0,1]. This is because the reported global sensitivity indices are only estimates of the true sensitivities, having been calculated from a finite number of samples. Monte Carlo variability generates estimates that are marginally different from their true values, such that when the true values are close to 0 or 1 the sensitivity estimates may fall just below 0 or above 1 [33]. Although increasing sample size typically resolves this small source of error (causing the difference between sensitivity estimates and their true values to converge to zero), it is possible to obtain this error for any sample size if a true sensitivity index is precisely 0 or 1. In practice then, the key to selecting an appropriate sample size involves both ensuring the convergence of the sensitivity estimates and deciding what an acceptable (negligible) difference (error bound) is. In the results presented here we considered estimates outside the range [0,1] by less than 0.01 acceptable (assuming the true sensitivity values were 0 or 1 as appropriate).

**Table 2:** First-order effect global sensitivity indices.

	Population density	Mean lifespan	Mean replacement period	Sociability scale parameter	Spatial scale parameter (*λ*)	Random number generator seed
Distribution	*U*(0.05,0.38)	*U*(2,4)	*U*(1,100)	$U\left(\frac{1}{30}\sqrt{0.5},\frac{1}{30}\sqrt{5}\right)$	*U*(0.1,1.0)	*U*{0,2^32^ − 1}
Milestone 4 (120 km)–binary	0.116	0.010	-0.008	**0.153**	0.013	0.008
Milestone 3 (90 km)–binary	0.115	0.009	-0.009	**0.152**	0.012	0.006
Milestone 2 (60 km)–binary	0.113	0.010	-0.008	**0.151**	0.012	0.006
Milestone 1 (30 km)–binary	0.110	0.010	-0.001	**0.145**	0.020	0.011
Number of dogs infected by the index case	0.070	0.012	0.005	**0.102**	0.005	0.003

Bold values indicate the input variable with highest sensitivity index for each respective outcome variable.

**Table 3:** Total effect global sensitivity indices.

	Population density	Mean lifespan	Mean replacement period	Sociability scale parameter	Spatial scale parameter (*λ*)	Random number generator seed
Distribution	*U*(0.05,0.38)	*U*(2,4)	*U*(1,100)	$U\left(\frac{1}{30}\sqrt{0.5},\frac{1}{30}\sqrt{5}\right)$	*U*(0.1,1.0)	*U*{0,2^32^ − 1}
Milestone 4 (120 km)–binary	**0.830**	0.650	0.663	0.565	0.297	0.668
Milestone 3 (90 km)–binary	**0.831**	0.652	0.665	0.565	0.298	0.669
Milestone 2 (60 km)–binary	**0.832**	0.655	0.668	0.561	0.303	0.673
Milestone 1 (30 km)–binary	**0.839**	0.675	0.687	0.546	0.326	0.694
Number of dogs infected by the index case	**0.898**	0.806	0.824	0.384	0.196	0.814

Bold values indicate the input variable with highest sensitivity index for each respective outcome variable.

For the binary outcomes indicating whether or not rabies percolated beyond each respective milestone distance, as well as the number of dogs infected by the index case, the wild dog sociability scale parameter and wild dog density were the first and second most influential input variables respectively (Table 2, Fig 6 Panels A and B). Specifically, the sociability scale parameter accounted for 15% of the variance in whether rabies percolated or not whilst wild dog density explained 11% of its variance. For the number of dogs infected by the index case these values declined slightly to 10% and 7% respectively. The importance of density, though, was more pronounced when input variable interactions were taken into account explaining over 83% of the variance in all outcomes (Table 3, Fig 6 Panels A and B).

## Discussion

Canine rabies typically persists in the context of an urban transmission cycle in which stray dogs and unvaccinated, free-roaming owned dogs account for a substantial proportion of the population [2, 8, 12, 27, 28, 40–42]. We have investigated the potentially unique scenario of canine rabies spreading in a sylvatic transmission cycle with the Australian wild dog as reservoir host. We estimate there is a 21% probability the incursion of a single sub-clinically infected dog and subsequent infection of one or more wild dogs will result in the sustained spatial transmission of rabies within the wild dog population, and on so doing spread at a mean (median) speed of 90 (67) km/year. Given current levels of uncertainty for wild dog ecology parameters, global sensitivity analysis indicates wild dog sociability and wild dog density are the first and second most influential parameters in determining whether a rabies epidemic will occur. Rate of spread, on the other hand, is governed by the spatial scale over which distance begins to affect wild dog contact rates.

In recent years, wildlife contact networks have attracted increasing levels of attention [43]. This is because individual contact behaviour (e.g. heterogeneity and territoriality) influences whether an epidemic can occur, how quickly a disease will spread, and the epidemic final size [44, 45]. Observation of real contact networks also facilitates the construction of contact network models with relevant properties, such that disease spread through populations can be simulated. These models can be used to identify factors driving transmission, predict patterns of disease spread, and design effective surveillance and intervention strategies [43]. Given the current absence of Australian wild dog contact network data, we employed a long-range percolation model from [22] to simulate the spread of rabies through Australia’s wild dog population. Importantly, the model naturally accommodates both heterogeneity (by assigning each dog its own sociability) and territoriality (through the spatial scale parameter *λ*). This model may be applied in a similar manner to other wildlife populations for which there is either no contact network information or the observation thereof is difficult. This is true even when the host population is not territorial (and the corresponding contact network is small-world in nature) since a sufficiently small value for *λ* would render each host capable of contacting every other host in the population.

Global sensitivity analysis is an increasingly popular approach to performing sensitivity analysis of epidemiological models [30]. This is because it complements a model’s ability to answer questions such as “What is the probability an epidemic will occur?” by identifying those factors most influential in determining its answer. One can therefore regard global sensitivity analysis as a tool which tells researchers ‘where to look’ and improves our understanding of the processes driving the spread of disease. That is to say, it highlights the parameters worth exploring further and measuring more accurately in the field such that improved model outcome predictions can be obtained. This is particularly useful when several model parameters have wide ranges reflecting large uncertainty or natural variation over space and time (as was the case for nearly all the wild dog ecology parameters in the current study) since a wide range of values for a particular parameter does not imply model outcomes will be sensitive to it (e.g. the mean replacement period). Given the parameter identified as most influential in determining whether a canine rabies epidemic will occur in Australian wild dogs (i.e. the sociability scale parameter) had a range defined as an order of magnitude (due to lack of published information) field data on wild dog movements and contact behaviour is glaringly missing. In this sense the main finding of the paper is consistent with [2]. We therefore recommend future research should focus on the observation of wild dog contact networks as this will go a long way to addressing this knowledge gap and could at the same time serve to confirm, or generate new hypotheses for, the assumed wild dog contact rate functional form (Eq 1).

The assumption that a wild dog’s sociability is equal to the square root of the area of land it traverses per day is a consequence of the wild dog contact rate proposed in Eq 1 (see S1 Appendix). Interestingly, the area of land traversed per day has the same units as those of the diffusion co-efficient *D* in a system of partial differential equations describing the spatial propagation of travelling infection waves. Furthermore, the velocity at which these travelling waves propagate is proportional to the square root of the diffusion co-efficient, $\sqrt{D}$ [46]. Thus our finding that wild dog sociability is a key parameter in the percolation of rabies through the wild dog population beyond milestone distances is consistent with established theory describing the spatial propagation of infection through a host population (e.g. fox rabies in Europe).

This work has some important caveats to bear in mind. The first is that rabies infection could modify wild dog behaviour in a manner significant for the sociability and spatial scale parameter, *λ*, values. Predicting exactly how these parameters, and possibly even the contact rate (Eq 1), might differ for rabid dogs, though, is not easy, especially given rabies presents in two forms, namely furious and dumb (paralytic) [2]. Future work should investigate the sensitivity of model outcomes to these factors by comparing results generated for alternative contact rate functions, as well as when the values for sociability and *λ* are conditional on wild dog disease status (i.e. furious or dumb).

A second caveat is that we assumed home ranges (nodes) remained unoccupied for an exponentially distributed period of time. In reality, the mean replacement period is likely to be a complex function of wild dog migration between home ranges (movements over and above normal everyday activity captured by the contact rate), seasonal birth rates, and wild dog density. Future research should include investigating whether incorporating more realistic wild dog demography (for which there is currently a paucity of knowledge, particularly for tropical ecosystems in northern Australia) will significantly alter the model outcomes and global sensitivity analysis results presented here. This will be especially important when comparing the risk of rabies spread in multiple locations, e.g. Arnhem Land and Cape York.

Thirdly, when simulating the spread of rabies we chose to fix the values of the canine rabies pathogenesis parameters as they are relatively well defined in the literature. Importantly, though, just because they were assigned fixed values does not mean model outcomes are insensitive to them. The pathogenesis parameters were assigned fixed values with the specific aim of identifying which ecological parameters (for which there is little known at present) are worth exploring further. The global sensitivity analysis results therefore need to be interpreted in this light.

A fourth caveat is that when parameterizing the model, we fixed the sociability shape parameter to an arbitrarily chosen value of 30. Choosing a larger value would have reduced the upper limit of the range of values from which the sociability scale parameter was sampled. This, in turn, would have reduced both the probability of percolation (Fig 4 Panel D) and the proportion of model outcome variance explained by the sociability scale parameter. If instead we had chosen a smaller value for the sociability shape parameter the opposite would be true. Consequently, accurately quantifying the parameters describing the distribution of wild dog sociabilities (from the area of land they traverse per day) will be important for future predictions of canine rabies spread in Australian wild dogs.

In order for a disease to become established each infectious dog must on average produce at least one subsequent infectious dog. To do this an infectious dog must first come into contact with enough other dogs such that it has sufficient opportunity to successfully infect at least one of them. When a dog in our model is sociable (i.e. when it covers a large area of land per unit time) and when wild dog density is high this requirement is satisfied, and therefore it is reasonable that wild dog sociability and density explain whether a rabies epidemic will occur. This interpretation agrees well with the finding that the number of dogs infected by the index case was also most sensitive to these two input variables.

To understand the nearly linear relationship between the rate of spread (time to milestone) and spatial scale parameter, *λ*, one has to first consider the implications of the territorial nature of wild dogs (represented in our model by the spatial scale parameter *λ*). When an infectious disease spreads in a territorial population (where the traditional random mixing assumption does not apply) a localized depletion of susceptible hosts may occur [22, 25]. Thus, in order for the disease to continue spreading, long-range transmission (host dispersal) is required such that it can ‘escape’ the localized outbreak. The further transmission (dispersal) occurs, however, the faster the disease will spread and the shorter the time taken until percolation. In the model presented here this occurs precisely when wild dogs traverse large distances (i.e. when *λ* is relatively small). This finding supports the hypothesis in [2] that rabies may spread faster in resource-poor areas, e.g. semi-arid or desert regions, where wild dogs traverse greater distances and have larger home ranges [47].

Rate of spread is an important outcome to consider since it determines the level of intervention required to contain an epidemic and bring about disease elimination once detected. Our estimates for the epidemic rate of spread are similar to, if not slightly above, the 20–80 km/year published in the literature for other sylvatic rabies virus strains and host populations [2, 48]. This may be because wild dogs are anatomically larger, with allometrically-scaled larger home ranges [49], and therefore genuinely more mobile (higher sociabilities, smaller *λ*) than raccoons, foxes, and badgers, but alternatively could just be a result of the wide parameter ranges we considered. In either case, the preparedness of Australia could be greatly improved by targeted field studies that aim to better understand wild dog movement and behaviour.

## Supporting Information

S1 Appendix (DOCX)

S1 Fig Number of dogs infected by the index case versus model input parameters. The mean number of dogs infected by the index case as a function of (A) wild dog density, (B) mean lifespan, (C) mean replacement period, (D) sociability scale parameter, (E) spatial scale parameter, *λ*, and (F) random number generator seed, given rabies percolates (blue, milestone 1) 30km, (green, milestone 2) 60 km, (red, milestone 3) 90 km, or (black, milestone 4) 120 km. The (magenta, all simulations) curve is the mean number of dogs infected by the index case irrespective of whether rabies percolates or not, equivalent to the basic reproduction number *R*_0_.
(PDF)

S2 Fig Number of nodes within milestone 1 versus model input parameters. The median number of nodes within milestone 1 as a function of (A) wild dog density, (B) mean lifespan, (C) mean replacement period, (D) sociability scale parameter, (E) spatial scale parameter, *λ*, and (F) random number generator seed, given rabies percolates (blue, milestone 1) 30 km, (green, milestone 2) 60 km, (red, milestone 3) 90 km, or (black, milestone 4) 120 km. The (magenta, all simulations) curve is the median number of nodes within milestone 1 irrespective of whether rabies percolates or not.
(PDF)

S3 Fig Number of dogs within milestone 1 before rabies introduction versus model input parameters. The median number of dogs within milestone 1 before rabies introduction as a function of (A) wild dog density, (B) mean lifespan, (C) mean replacement period, (D) sociability scale parameter, (E) spatial scale parameter, *λ*, and (F) random number generator seed, given rabies percolates (blue, milestone 1) 30 km, (green, milestone 2) 60 km, (red, milestone 3) 90 km, or (black, milestone 4) 120 km. The (magenta, all simulations) curve is the median number of dogs within milestone 1 before rabies introduction irrespective of whether rabies percolates or not.
(PDF)

S4 Fig Reduction in wild dog density within milestone 1 versus model input parameters. The median reduction in wild dog density within milestone 1 as a function of (A) wild dog density, (B) mean lifespan, (C) mean replacement period, (D) sociability scale parameter, (E) spatial scale parameter, *λ*, and (F) random number generator seed, given rabies percolates beyond milestone 4 (120 km).
(PDF)

S5 Fig Proportion of infected (exposed) dogs within milestone 1 versus model input parameters. The median proportion of infected (exposed) dogs within milestone 1 as a function of (A) wild dog density, (B) mean lifespan, (C) mean replacement period, (D) sociability scale parameter, (E) spatial scale parameter, *λ*, and (F) random number generator seed, given rabies percolates beyond milestone 4 (120 km).
(PDF)

S6 Fig Proportion of infectious dogs within milestone 1 versus model input parameters. The median proportion of infectious dogs within milestone 1 as a function of (A) wild dog density, (B) mean lifespan, (C) mean replacement period, (D) sociability scale parameter, (E) spatial scale parameter, *λ*, and (F) random number generator seed, given rabies percolates beyond milestone 4 (120 km).
(PDF)

## References

[1] World Health Organization. WHO expert consultation on rabies: second report. Geneva: World Health Organ Tech Rep Ser, 2013. 24069724

[2] Sparkes J, Fleming PJ, Ballard G, Scott-Orr H, Durr S, Ward MP. Canine rabies in Australia: a review of preparedness and research needs. Zoonoses Public Health. 2014;62:237–53. 10.1111/zph.12142 24934203

[3] World Health Organization. Rabies vaccines: WHO position paper. Wkly Epidemiol Rec. 2010;85(32).

[4] Paweska JT, Blumberg LH, Liebenberg C, Hewlett RH, Grobbelaar AA, Leman PA, et al Fatal human infection with rabies-related Duvenhage virus, South Africa. Emerg Infect Dis. 2006;12(12):1965–7. 10.3201/eid1212.060764 17326954 PMC3291369

[5] World Health Organization. Rabies fact sheet no 99: World Health Organization; 2014 [6 June 2015]. Available from: http://www.who.int/mediacentre/factsheets/fs099/en/.

[6] Smith JS. New aspects of rabies with emphasis on epidemiology, diagnosis, and prevention of the disease in the United States. Clin Microbiol Rev. 1996;9(2):166–&. 8964034 10.1128/cmr.9.2.166 PMC172889

[7] Wandeler AI, Bingham J, Meslin FX. Dogs and rabies In: Macpherson CNL, Meslin FX, Wandeler AI, editors. Dogs, Zoonoses and Public Health. Wallingford, UK: CABI; 2013.

[8] Hutabarat T, Geong M, Newsome A, Ruben A, Cutter S. Rabies and dog ecology in Flores. Urban Animal Management Conference Proceedings ACIAR Australia, 2003.

[9] Putra AAG, Hampson K, Girardi J, Hiby E, Knobel D, Wayan Mardiana I, et al Response to a rabies epidemic, Bali, Indonesia, 2008–2011. Emerg Infect Dis. 2013;19(4):648–51. 10.3201/eid1904.120380 23632033 PMC3647408

[10] Fleming PJ, Allen BL, Ballard G, Allen LR. Wild dog ecology, impacts and management in northern Australian cattle enterprises: a review with recommendations for RD&E investments. 2012.

[11] Tenzin Ward MP. Review of rabies epidemiology and control in South, South East and East Asia: past, present and prospects for elimination. Zoonoses Public Health. 2012;59(7):451–67. 23180493 10.1111/j.1863-2378.2012.01489.x

[12] Putra AAG, Gunata I, Dharma D, Scott-Orr H, editors. Rabies on the move in Indonesia: incursion into Bali and response. Proceedings of the 12th Symposium of the International Society for Veterinary Epidemiology and Economics, 2009; Durban, South Africa.

[13] Ward MP, Hernandez-Jover M. A generic rabies risk assessment tool to support surveillance. Prev Vet Med. 2015;120(1):4–11. 10.1016/j.prevetmed.2014.11.005 25466214

[14] Brookes VJ, Ward MP. Expert opinion to identify high-risk entry routes of canine rabies into Papua New Guinea. Zoonoses Public Health. 2016:n/a-n/a. 10.1111/zph.12284 27362859

[15] Durr S, Ward MP. Roaming behaviour and home range estimation of domestic dogs in Aboriginal and Torres Strait Islander communities in northern Australia using four different methods. Prev Vet Med. 2014;117(2):340–57. 10.1016/j.prevetmed.2014.07.008 25096735

[16] Sparkes J, Kortner G, Ballard G, Fleming PJS, Brown WY. Effects of sex and reproductive state on interactions between free-roaming domestic dogs. PloS One. 2014;9(12):13. 10.1371/journal.pone.0116053 PMC4277450 25541983

[17] Allen BL, West P. Influence of dingoes on sheep distribution in Australia. Aust Vet J. 2013;91(7):261–7. 10.1111/avj.12075 23782018

[18] Fleming PJS, Allen BL, Allen LR, Ballard G, Bengsen AJ, Gentle MN, et al Management of wild canids in Australia: free-ranging dogs and red foxes In: Glen AS, Dickman CR, editors. Carnivores of Australia: past, present and future. Collingwood, Melbourne: CSIRO Publishing; 2014 p. 105–49.

[19] Sparkes J, Ballard G, Fleming PJ, Brown W. Social, conservation and economic implications of rabies in Australia. Aust Zool. 2014.

[20] Anderson RM, May RM. Infectious diseases of humans: dynamics and control. Oxford: Oxford University Press; 1991.

[21] Durr S, Ward MP. Development of a novel rabies simulation model for application in a non-endemic environment. PLoS Negl Trop Dis. 2015;9(6):22. 10.1371/journal.pntd.0003876 PMC4482682 26114762

[22] Davis SA. Percolation on a spatial network with individual heterogeneity as a model for disease spread among animal host populations. 19th International Congress on Modelling and Simulation. 2011:905–11.

[23] Fleming PJ, Bomford M. Managing the impacts of dingoes and other wild dogs: Bureau of Rural Sciences; 2001.

[24] Corbett LK. The dingo in Australia and Asia. Marleston: JB Books Australia; 2001.

[25] Davis S, Trapman P, Leirs H, Begon M, Heesterbeek JA. The abundance threshold for plague as a critical percolation phenomenon. Nature. 2008;454(7204):634–7. 10.1038/nature07053 18668107

[26] Grimmett G. Percolation. Berlin: Springer-Verlag; 1999.

[27] Hampson K, Dushoff J, Cleaveland S, Haydon DT, Kaare M, Packer C, et al Transmission dynamics and prospects for the elimination of canine rabies. PLoS Biol. 2009;7(3):462–71. 10.1371/journal.pbio.1000053 PMC2653555 19278295

[28] Hampson K, Dushoff J, Bingham J, Bruckner G, Ali YH, Dobson A. Synchronous cycles of domestic dog rabies in sub-Saharan Africa and the impact of control efforts. Proc Natl Acad Sci U S A. 2007;104(18):7717–22. PubMed Central PMCID: PMC1863501. 10.1073/pnas.0609122104 17452645 PMC1863501

[29] Carroll MJ, Singer A, Smith GC, Cowan DP, Massei G. The use of immunocontraception to improve rabies eradication in urban dog populations. Wildl Res. 2010;37(8):676–87.

[30] Wu JY, Dhingra R, Gambhir M, Remais JV. Sensitivity analysis of infectious disease models: methods, advances and their application. J R Soc Interface. 2013;10(86):14. 10.1098/rsif.2012.1018 PMC3730677 23864497

[31] Saltelli A. Making best use of model evaluations to compute sensitivity indices. Comput Phys Commun. 2002;145(2):280–97.

[32] Cannavó F. Sensitivity analysis for volcanic source modeling quality assessment and model selection. Comput Geosci. 2012;44:52–9.

[33] Archer GEB, Saltelli A, Sobol IM. Sensitivity measures, ANOVA-like techniques and the use of bootstrap. J Stat Comput Simul. 1997;58(2):99–120.

[34] Stephens D, Wilton AN, Fleming PJS, Berry O. Death by sex in an Australian icon: a continent-wide survey reveals extensive hybridization between dingoes and domestic dogs. Mol Ecol. 2015;24(22):5643–56. 10.1111/mec.13416 26514639

[35] Foggin CM. Rabies and rabies-related viruses in Zimbabwe: historical, virological and ecological aspects [Dissertation]. Harare: University of Zimbabwe; 1988.

[36] Tepsumethanon V, Lumlertdacha B, Mitmoonpitak C, Sitprija V, Meslin FX, Wilde H. Survival of naturally infected rabid dogs and cats. Clin Infect Dis. 2004;39(2):278–80. 10.1086/421556 15307040

[37] Coleman PG, Dye C. Immunization coverage required to prevent outbreaks of dog rabies. Vaccine. 1996;14(3):185–6. 8920697 10.1016/0264-410x(95)00197-9

[38] Fleming PJS, Ballard G, Newsome TM. Unpublished data.

[39] James CD, Landsberg J, Morton Sr. Provision of watering points in the Australian arid zone: a review of effects on biota. J Arid Environ. 1999;41(1):87–121.

[40] Eng TR, Fishbein DB, Talamante HE, Hall DB, Chavez GF, Dobbins JG, et al Urban epizootic of rabies in Mexico: epidemiology and impact of animal bite injuries. Bull World Health Organ. 1993;71(5):615–24. 8261565 PMC2393488

[41] Morters MK, Restif O, Hampson K, Cleaveland S, Wood JLN, Conlan AJK. Evidence-based control of canine rabies: a critical review of population density reduction. J Anim Ecol. 2014;83(5):1244–. 23004351 10.1111/j.1365-2656.2012.02033.x PMC3579231

[42] Hampson K, Dobson A, Kaare M, Dushoff J, Magoto M, Sindoya E, et al Rabies exposures, post-exposure prophylaxis and deaths in a region of endemic canine rabies. PLoS Negl Trop Dis. 2008;2(11):e339 PubMed Central PMCID: PMC2582685. 10.1371/journal.pntd.0000339 19030223 PMC2582685

[43] Craft ME. Infectious disease transmission and contact networks in wildlife and livestock. Philos Trans R Soc Lond B Biol Sci. 2015;370(1669):12. 10.1098/rstb.2014.0107 PMC4410373 25870393

[44] Keeling MJ, Eames KTD. Networks and epidemic models. J R Soc Interface. 2005;2(4):295–307. 10.1098/rsif.2005.0051 16849187 PMC1578276

[45] Danon L, Ford AP, House T, Jewell CP, Keeling MJ, Roberts GO, et al Networks and the epidemiology of infectious disease. Interdiscip Perspect Infect Dis. 2011. 10.1155/2011/284909 PMC3062985 21437001

[46] Panjeti VG, Real LA. Mathematical models for rabies. Adv Virus Res. 2011;79:377–95. 10.1016/B978-0-12-387040-7.00018-4 21601056

[47] Newsome TM, Ballard GA, Dickman CR, Fleming PJS, Van De Ven R. Home Range, activity and sociality of a top predator, the dingo: a test of the Resource Dispersion Hypothesis. Ecography. 2013;36(8):914–25.

[48] Lemey P, Rambaut A, Welch JJ, Suchard MA. Phylogeography takes a relaxed random walk in continuous space and time. Mol Biol Evol. 2010;27(8):1877–85. 10.1093/molbev/msq067 20203288 PMC2915639

[49] Hendriks AJ, Willers BJC, Lenders HJR, Leuven RSEW. Towards a coherent allometric framework for individual home ranges, key population patches and geographic ranges. Ecography. 2009;32(6):929–42.

