# Supplementary material for: Predicted Spatial Spread of Canine Rabies in Australia
Source: PLoS Negl Trop Dis. 2017 Jan 23;11(1):e0005312. doi: 10.1371/journal.pntd.0005312 (PMC5289603; doi:10.1371/journal.pntd.0005312)
Supplement: S1 Appendix — (DOCX) [file pntd.0005312.s001.docx]

# S1 Appendix

## Deriving the density of nodes to be distributed

Before the introduction of rabies, nodes, representing the centroids of potential wild dog home ranges, are either occupied by susceptible wild dogs or unoccupied (Fig). If we assume that:

1. Only two demographic processes are at work prior to rabies introduction, namely death due to natural causes (rendering occupied nodes unoccupied) and replacement (rendering unoccupied nodes re-occupied by susceptible wild dogs),
2. The lifespan of wild dogs and the replacement period are both exponentially distributed (with constant mean values), and
3. The density of susceptible wild dogs (occupied nodes) prior to rabies introduction is at a non-trivial equilibrium,

then an analytic relationship between the density of nodes and the density of susceptible wild dogs (occupied nodes) can be derived. To do this, we let

$\Omega\left( t \right)$ = the density of occupied nodes (susceptible wild dogs) at time $t$,

$\Upsilon\left( t \right)$ = the density of unoccupied nodes at time $t$,

$N\left( t \right)$ = the density of nodes (occupied and unoccupied) at time $t$

= $\Omega\left( t \right)+\Upsilon\left( t \right)$, (S1)

$\mu$ = the natural mortality rate

= 1/(mean wild dog lifespan), and

$\rho$ = the replacement rate

= 1/(mean replacement period).

From assumptions 1 and 2 it follows that the behaviour over time of the density of occupied nodes (for a landscape of fixed area) is described by

$\frac{d\Omega}{dt}=\rho\Upsilon-\mu\Omega.$ (S2)

Substituting Equation S1 into S2 yields

$\frac{d\Omega}{dt}=\rho N-\left( \rho+\mu\right)\Omega.$ (S3)

At equilibrium (assumption 3) we have that $d\Omega/dt=0$, which implies

$N=\left( \frac{\rho+\mu}{\rho} \right)\Omega.$ (S4)

Equation S4 is the relationship we set out to derive and can be used to calculate the density of nodes to be distributed across the landscape at the start of a simulation (prior to rabies introduction) provided the mean wild dog lifespan, mean replacement period, and susceptible wild dog density for that simulation are all known. Also worth noting is that node density is equal to the density of susceptible wild dogs multiplied by a factor $1/\pi$, where $\pi=\left( \frac{\rho}{\rho+\mu} \right)$ is the proportion of nodes occupied by susceptible wild dogs. We made use of this insight, together with Equation S4, to implement each simulation as follows:

1. Values for each of the model input variables (including mean wild dog lifespan, mean replacement period, and susceptible wild dog density) were sampled from their respective distributions (see Global sensitivity analysis section in main text),
2. The samples generated in 1 were used to calculate the number of nodes to be distributed across the simulated 250 km x 125 km landscape using Equation S4,
3. Nodes were uniformly randomly distributed across the landscape until the node density calculated in 2 was reached,
4. Each node in 3 was occupied by a susceptible wild dog with probability $\pi$ and each dog assigned its own unique sociability,
5. The susceptible wild dog closest to the centre of the upper boundary of the landscape was infected with rabies (the index case) to emulate rabies incursion along the northern coast of Australia, and
6. Rabies transmission between wild dogs was simulated until either no more dogs were infected or rabies had percolated beyond the fourth milestone distance (120 km from the index case).


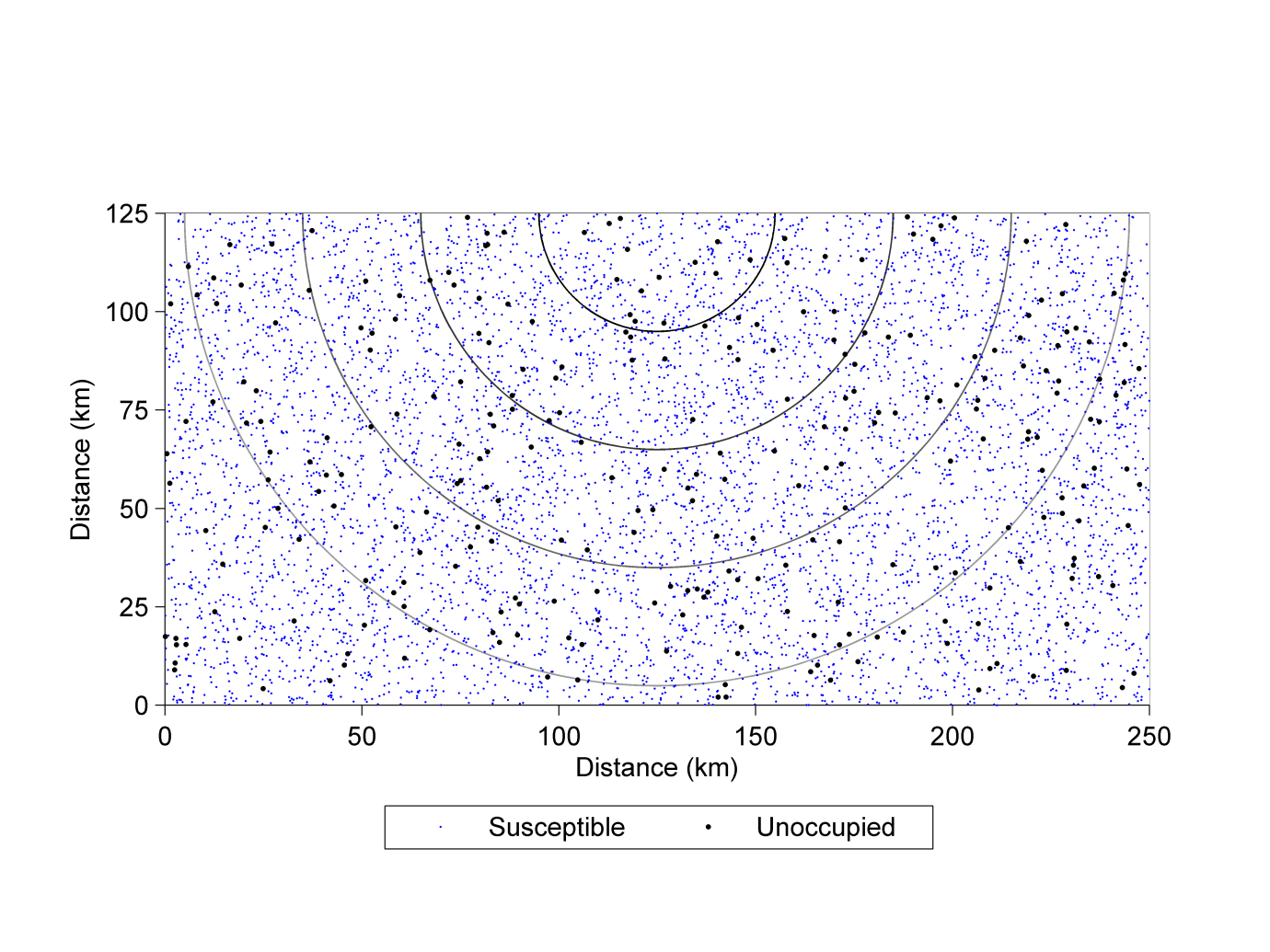


**Fig. The distribution of susceptible wild dogs (occupied nodes) prior to canine rabies introduction.**

Each node, representing the centroid of a potential wild dog home range, is either (blue) occupied by a susceptible wild dog or (bold black) unoccupied. An occupied node’s location corresponds to the average position of the dog that occupies the home range. We assume only two demographic processes are at work prior to the introduction of a sub-clinically infected (exposed) index case, namely death due to natural causes (rendering occupied nodes unoccupied) and replacement (rendering unoccupied nodes re-occupied by susceptible wild dogs).

## Wild dog contact rate dimensional analysis

To illustrate that a wild dog’s sociability, $x_{i}$, is related to the area of land it traverses per day we perform dimensional analysis of the wild dog contact rate, $k_{ij}$, proposed in Equation 1 of the main text and repeated here for ease of reference:

$k_{ij}=\lambda^{2}e^{-\lambda s_{ij}}x_{i}x_{j}.$ (S5)

A variable enclosed in square brackets, e.g. $\left[ x_{i} \right]$, will be used to denote the variable’s dimension, whilst $T$ and $D$ will be used to indicate the dimensions of time and distance respectively.

First, we observe that $k_{ij}$ is a rate and therefore must have dimension $\left[ k_{ij} \right]=T^{-1}$. Next, because $s_{ij}$ is the Euclidian distance between the mean positions (nodes) of dogs $i$ and $j$ it must have dimension $\left[ s_{ij} \right]=D$. Also, the exponent on the right-hand side of Equation S5 must be dimensionless which implies the units of $\lambda$ and $s_{ij}$ cancel out, i.e. $\left[ \lambda\right]=\frac{1}{\left[ s_{ij} \right]}=D^{-1}$. Rearranging Equation S5 and recognizing $\left[ x_{j} \right]=\left[ x_{i} \right]$ it follows that

$\left[ x_{i} \right]^{2}=\frac{\left[ k_{ij} \right]}{\left[ \lambda\right]^{2}}=\frac{D^{2}}{T},$ (S6)

which then gives

$\left[ x_{i} \right]=\sqrt{\frac{D^{2}}{T}}.$ (S7)

If we consider the radicand under the square root, we recognize from its dimension $\left( {D^{2}}/T \right)$ that it represents area per unit time. Equation S7 therefore tells us that a wild dog which traverses a large area of land per unit time is one that is highly sociable. This is appealing because it is natural to expect a dog which covers a large area of land per unit time to encounter more dogs during some time interval than one which only covers a small area of land per unit time. An implication of this result worth noting is that just because a wild dog has a small *home range* does not mean it is not sociable. If, for example, it were to traverse its small home range quite frequently then it could still encounter other dogs on a regular basis, even if they happen to be the same dogs on multiple occasions. Thus, we understand that a wild dog’s propensity for contacting other dogs (i.e. its sociability) is related to the area of land it traverses per unit time rather than the size of its home range.

Lastly, we note that for the particular units implemented in our model ($D$ ~ km, $T$ ~ day) the units of a wild dog’s sociability are km/day^0.5^.

## Global sensitivity analysis – technical details

To quantify the sensitivity of model outcomes to each input variable’s uncertainty we calculated Sobol’s indices by implementing the Monte Carlo procedure proposed by Saltelli [1]. Before describing the key steps involved we briefly review the theory behind Sobol’s indices and in particular the formulas estimated during the Monte Carlo procedure.

Consider a generic model $z=f\left( y_{1},y_{2},\ldots,y_{k} \right)$ with $k$ independent random input variables that have joint probability density function $p\left( y_{1},y_{2},\ldots,y_{k} \right)=\prod_{i=1}^{k} p_{i}\left( y_{i} \right)$. Also, consider $\boldsymbol{v}$, a subset of the input variables, and $\boldsymbol{u}$ its complement such that $\left\{ \boldsymbol{v},\boldsymbol{u} \right\}=\left\{ y_{1},y_{2},\ldots,y_{k} \right\}$. The sensitivity of outcome variable $z$ with respect to subset $\boldsymbol{v}$ is well described by two sensitivity indices, namely the first-order effect and total effect, $S_{\boldsymbol{v}}$ and $S_{\boldsymbol{v}}^{T}$ respectively. Of particular interest to us is when $\boldsymbol{v}$ includes only a single input variable, e.g. $\boldsymbol{v}=\left\{ y_{j} \right\}$. In this case we say $S_{\boldsymbol{v}}$ is the first-order effect of input variable $y_{j}$ and write

$S_{\boldsymbol{v}}=S_{j}=\left( \frac{U_{j}-E^{2}\left( z \right)}{V\left( z \right)} \right),$ (S8)

where

$$E^{2}\left( z \right)=\left\{ \iint\cdots\int f\left( y_{1},y_{2},\ldots,y_{k} \right)\prod_{i=1}^{k} p_{i}\left( y_{i} \right)dy_{i} \right\}^{2}$$

(S9)

is the square of the mean value of $z$,

$$V\left( z \right)=\iint\cdots\int f^{2}\left( y_{1},y_{2},\ldots,y_{k} \right)\prod_{i=1}^{k} p_{i}\left( y_{i} \right)dy_{i}-E^{2}\left( z \right)$$

(S10)

is the variance of $z$, and

$$U_{j}=\iint\cdots\int f\left( y_{1},y_{2},\ldots,y_{j},\ldots,y_{k} \right)f\left( y_{1}^{'},y_{2}^{'},\ldots,y_{j},\ldots,y_{k}^{'} \right)\prod_{i=1}^{k} p_{i}\left( y_{i} \right)dy_{i}\prod_{\begin{aligned} i=1 \\ i\neq j \end{aligned}}^{k} p_{i}\left( y_{i}^{'} \right)dy_{i}^{'}$$

(S11)

is the mean value of a function $F$ of $\left( 2k-1 \right)$ variables defined as

$$F\left( y_{1},y_{2},\ldots,y_{j},\ldots,y_{k},y_{1}^{'},y_{2}^{'},\ldots,y_{j-1}^{'},y_{j+1}^{'},\ldots,y_{k}^{'} \right)=f\left( y_{1},y_{2},\ldots,y_{j},\ldots,y_{k} \right)f\left( y_{1}^{'},y_{2}^{'},\ldots,y_{j},\ldots,y_{k}^{'} \right).$$

(S12)

Important to note is that in Equations S11 and S12:

1. The primed random input variables have the same distributions as their non-primed counterparts, i.e. $y_{i}$ and $y_{i}^{'}$ have the same distribution, but their values are independent, and
2. There is no random variable $y_{j}^{'}$ (or equivalently one can think of $y_{j}^{'}$ as always taking the same value as random variable $y_{j}$).

Similar to the first-order effect, when $\boldsymbol{v}=\left\{ y_{j} \right\}$, we say $S_{\boldsymbol{v}}^{T}$ is the total effect of input variable $y_{j}$ and write

${S_{\boldsymbol{v}}^{T}=S}_{j}^{T}=\left( 1-S_{\neg j} \right)=\left( 1-S_{\boldsymbol{u}} \right),$ (S13)

where

$S_{\neg j}=S_{\boldsymbol{u}}=\left( \frac{U_{\boldsymbol{u}}-E^{2}\left( z \right)}{V\left( z \right)} \right)$ (S14)

is the first-order effect for the set of all input variables excluding $y_{j}$, namely subset $\boldsymbol{u}$, and

$$U_{\boldsymbol{u}}=\iint\cdots\int f\left( y_{1},y_{2},\ldots,y_{j},\ldots,y_{k} \right)f\left( y_{1},y_{2},\ldots,y_{j}^{'},\ldots,y_{k} \right)\prod_{i=1}^{k} p_{i}\left( y_{i} \right)dy_{i}p_{j}\left( y_{j}^{'} \right)dy_{j}^{'}$$

(S15)

is the mean value of a function $G$ of $\left( k+1 \right)$ input variables defined as

$$G\left( y_{1},y_{2},\ldots,y_{j},\ldots,y_{k},y_{j}^{'} \right)=f\left( y_{1},y_{2},\ldots,y_{j},\ldots,y_{k} \right)f\left( y_{1},y_{2},\ldots,y_{j}^{'},\ldots,y_{k} \right).$$

(S16)

From Equations S8 – S16 it follows that to calculate the first-order and total effects of an input variable $y_{j}$ on an outcome variable $z$ one must evaluate four integrals (Equations S9 – S11 and S15) that are the mean values of closely related functions, e.g. $F$ and $G$. In a Monte Carlo framework this is easily done by implementing the following procedure proposed by Saltelli [1]. First, distributions for each of the $k$ input variables are defined. Next, two sample matrices

$\mathbf{M}=\left( \begin{matrix} y_{11} & y_{12} & \cdots& y_{1k} \\ y_{21} & y_{22} & \cdots& y_{2k} \\ \cdots& \cdots& \cdots& \cdots\\ y_{n1} & y_{n2} & \cdots& y_{nk} \end{matrix} \right)$ and $\mathbf{M}^{\mathbf{'}}=\left( \begin{matrix} y_{11}^{'} & y_{12}^{'} & \cdots& y_{1k}^{'} \\ y_{21}^{'} & y_{22}^{'} & \cdots& y_{2k}^{'} \\ \cdots& \cdots& \cdots& \cdots\\ y_{n1}^{'} & y_{n2}^{'} & \cdots& y_{nk}^{'} \end{matrix} \right)$

are generated by sampling each input variable distribution a total of $2n$ times ($n$ times for each matrix). Thereafter, both $E^{2}\left( z \right)$ and $V\left( z \right)$ are calculated from the products of the values of $z$ computed on the rows (sample vectors) of $\mathbf{M}$ and $\mathbf{M}^{\mathbf{'}}$ as follows

$$\hat{E}^{2}\left( z \right)=\frac{1}{n}\sum_{r=1}^{n} f\left( y_{r1},y_{r2},\ldots,y_{rk} \right)f\left( y_{r1}^{'},y_{r2}^{'},\ldots,y_{rk}^{'} \right)$$

(S17)

$$\hat{V}\left( z \right)=\frac{1}{n}\sum_{r=1}^{n} f\left( y_{r1},y_{r2},\ldots,y_{rk} \right)f\left( y_{r1},y_{r2},\ldots,y_{rk} \right)-\hat{E}^{2}\left( z \right),$$

(S18)

where $n$ is the sample size of the Monte Carlo estimates. Following this, Monte Carlo estimates for $U_{j}$ and $U_{\boldsymbol{u}}$ are obtained from

$$\hat{U}_{j}=\frac{1}{n}\sum_{r=1}^{n} f\left( y_{r1},y_{r2},\ldots,y_{rk} \right)f\left( y_{r1}^{'},y_{r2}^{'},\ldots,y_{r\left( j-1 \right)}^{'},y_{rj},y_{r\left( j+1 \right)}^{'},\ldots,y_{rk}^{'} \right)$$

(S19)

$$\hat{U}_{\boldsymbol{u}}=\frac{1}{n}\sum_{r=1}^{n} f\left( y_{r1},y_{r2},\ldots,y_{rk} \right)f\left( y_{r1},y_{r2},\ldots,y_{r\left( j-1 \right)},y_{rj}^{'},y_{r\left( j+1 \right)},\ldots,y_{rk} \right),$$

(S20)

where the inputs for the second factor in the products of the two equations are the rows (sample vectors) of two additional sample matrices,

$$\mathbf{N}_{j}=\left( \begin{matrix} y_{11}^{'} & y_{12}^{'} & \cdots& y_{1j} & \cdots& y_{1k}^{'} \\ y_{21}^{'} & y_{22}^{'} & \cdots& y_{2j} & \cdots& y_{2k}^{'} \\ \cdots& \cdots& \cdots& \cdots& \cdots& \cdots\\ y_{n1}^{'} & y_{n2}^{'} & \cdots& y_{nj} & \cdots& y_{nk}^{'} \end{matrix} \right)$$

and

$$\mathbf{N}_{\boldsymbol{u}}=\mathbf{N}_{\neg j}=\left( \begin{matrix} y_{11} & y_{12} & \cdots& y_{1j}^{'} & \cdots& y_{1k} \\ y_{21} & y_{22} & \cdots& y_{2j}^{'} & \cdots& y_{2k} \\ \cdots& \cdots& \cdots& \cdots& \cdots& \cdots\\ y_{n1} & y_{n2} & \cdots& y_{nj}^{'} & \cdots& y_{nk} \end{matrix} \right)$$

respectively, constructed from combinations of the columns of $\mathbf{M}$ and $\mathbf{M}^{\mathbf{'}}$.

In closing we note that, because matrices $\mathbf{N}_{j}$ and $\mathbf{N}_{\neg j}$ are specific to input variable $y_{j}$, for a model with $k$ input variables a total of $2\left( k+1 \right)$ sample matrices of size $\left( n\times k \right)$ need to be constructed to obtain a full set of first-order and total effect sensitivity indices. This, in turn, requires the implementation of $2n\left( k+1 \right)$ simulations, one for each row of each matrix.

## References

1. Saltelli, A., *Making best use of model evaluations to compute sensitivity indices.* Comput Phys Commun, 2002. **145**(2): p. 280-297.
