# Supplementary figures and images for: Predicted Spatial Spread of Canine Rabies in Australia
Source: PLoS Negl Trop Dis. 2017 Jan 23;11(1):e0005312. doi: 10.1371/journal.pntd.0005312 (PMC5289603; doi:10.1371/journal.pntd.0005312)

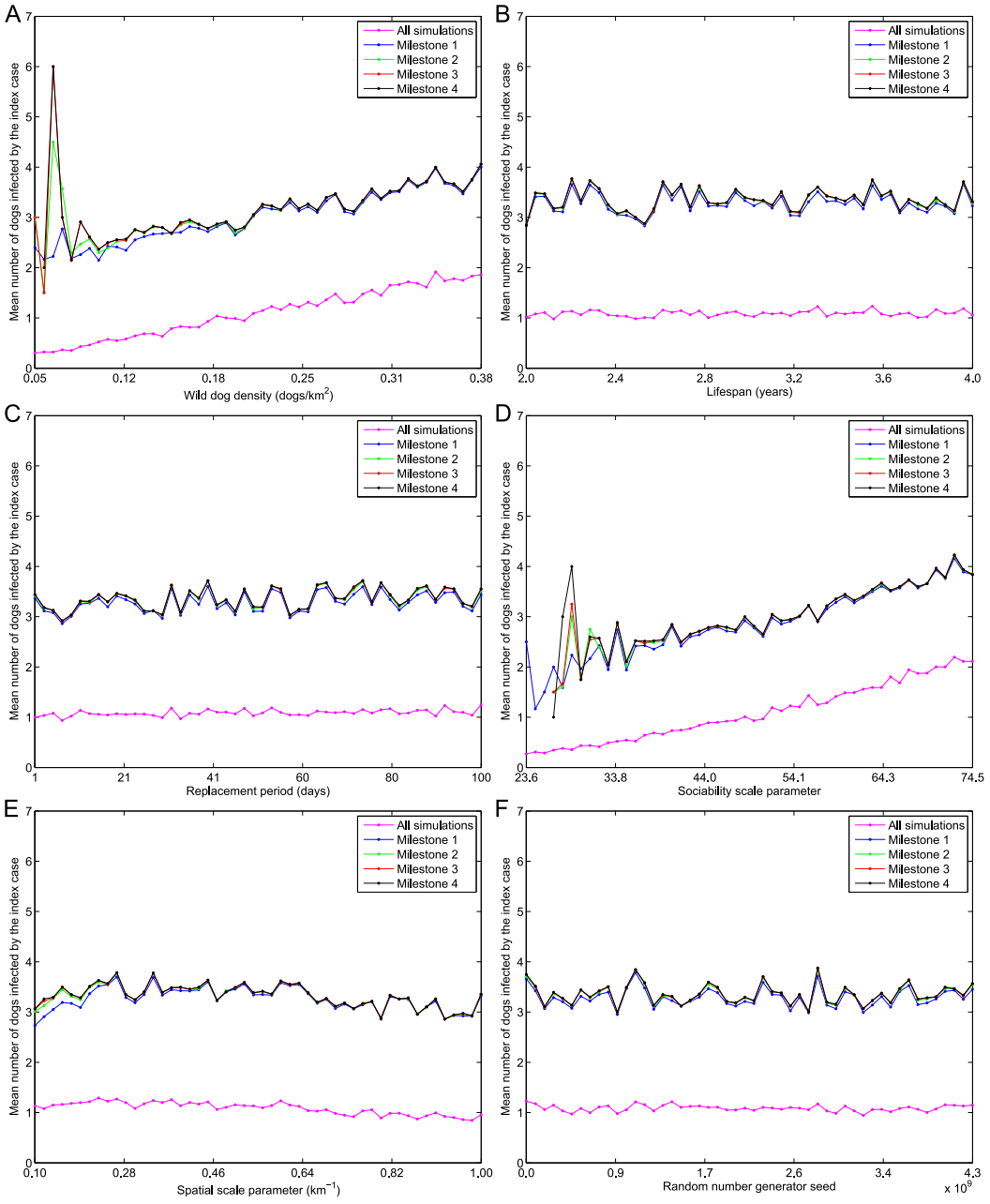

Supplement: S1 Fig — The mean number of dogs infected by the index case as a function of (A) wild dog density, (B) mean lifespan, (C) mean replacement period, (D) sociability scale parameter, (E) spatial scale parameter, λ, and (F) random number generator seed, given rabies percolates (blue, milestone 1) 30km, (green, milestone 2) 60 km, (red, milestone 3) 90 km, or (black, milestone 4) 120 km. The (magenta, all simulations) curve is the mean number of dogs infected by the index case irrespective of whether rabies percolates or not, equivalent to the basic reproduction number R0. (PDF) [file pntd.0005312.s002.pdf]

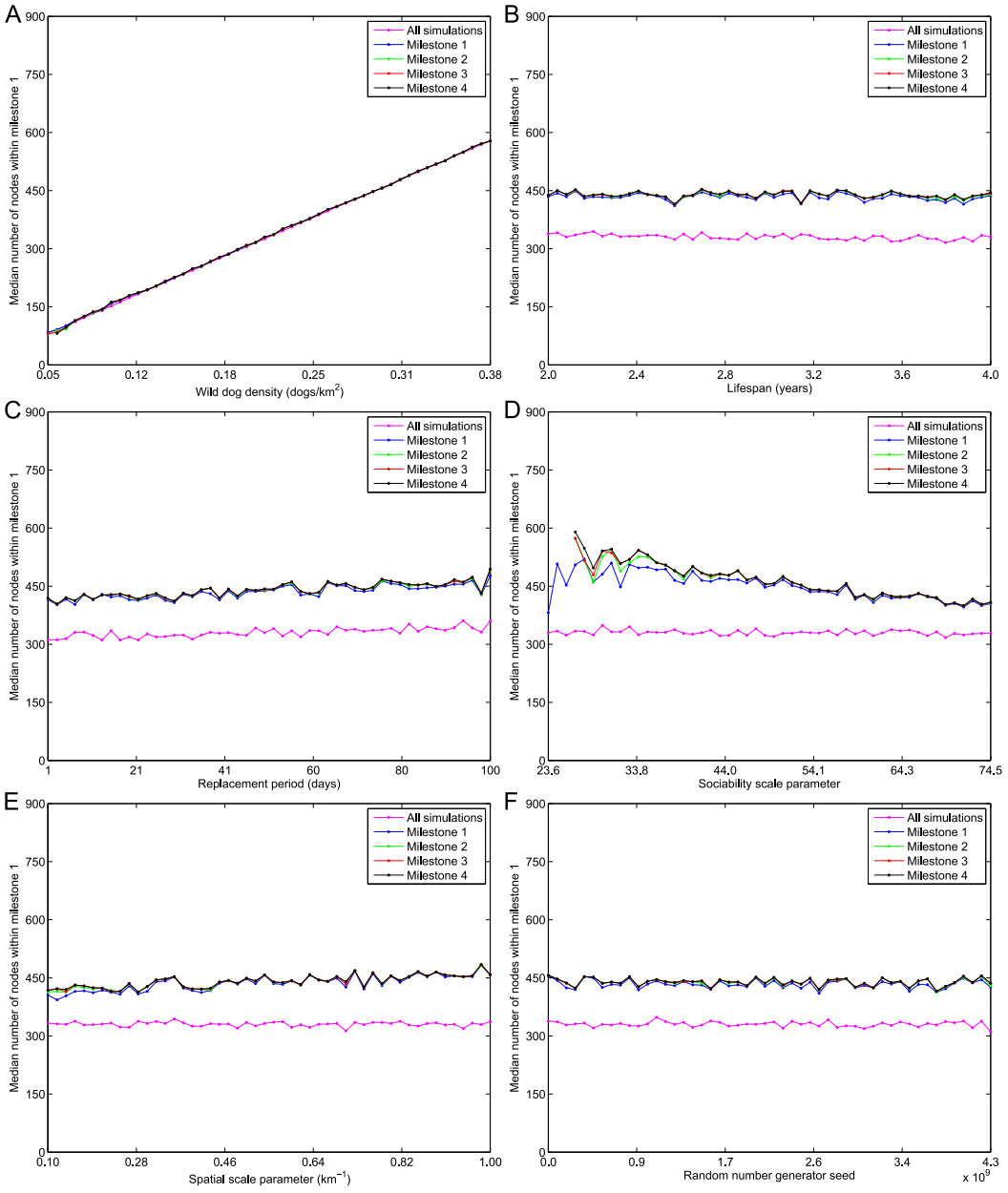

Supplement: S2 Fig — The median number of nodes within milestone 1 as a function of (A) wild dog density, (B) mean lifespan, (C) mean replacement period, (D) sociability scale parameter, (E) spatial scale parameter, λ, and (F) random number generator seed, given rabies percolates (blue, milestone 1) 30 km, (green, milestone 2) 60 km, (red, milestone 3) 90 km, or (black, milestone 4) 120 km. The (magenta, all simulations) curve is the median number of nodes within milestone 1 irrespective of whether rabies percolates or not. (PDF) [file pntd.0005312.s003.pdf]

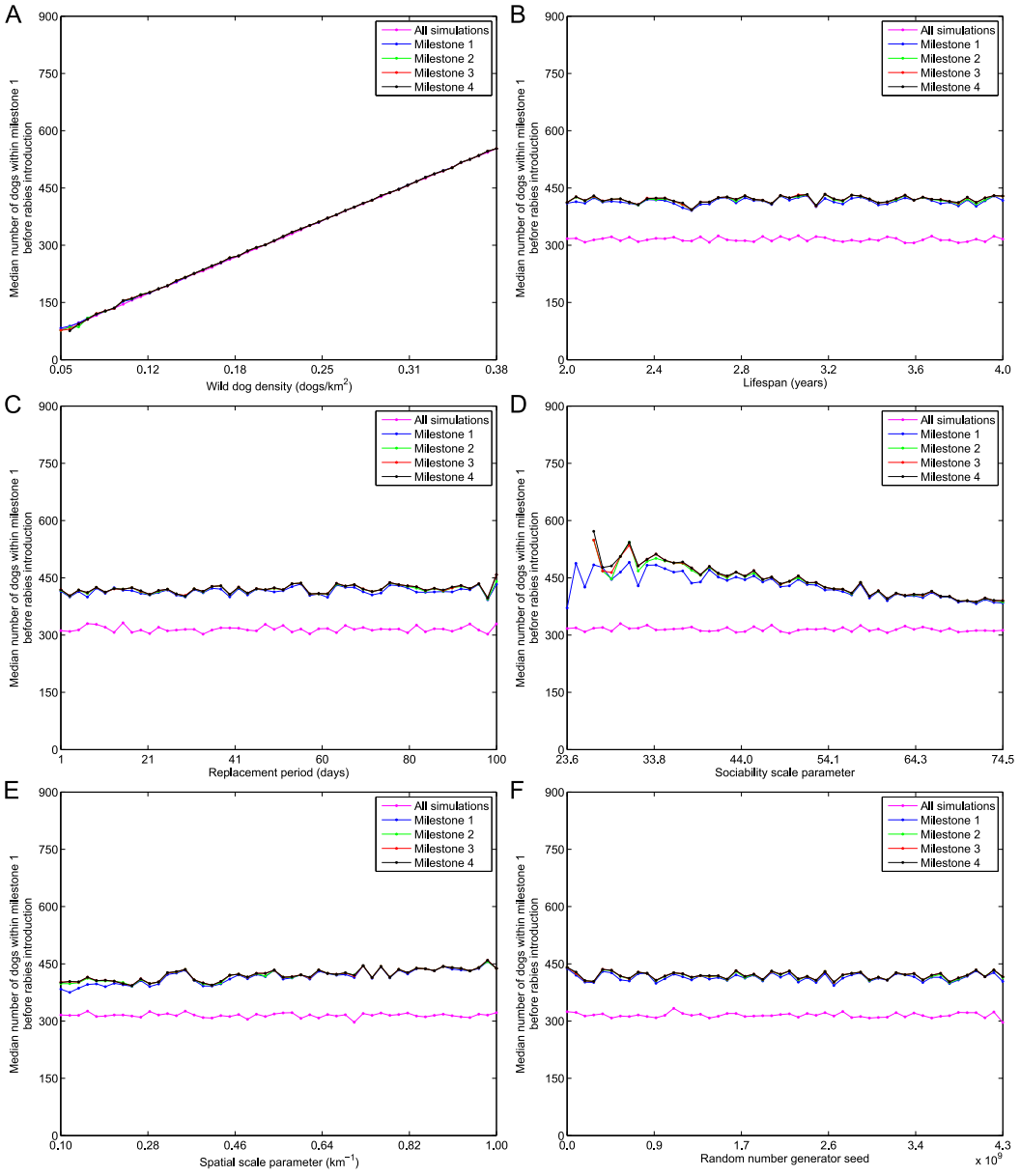

Supplement: S3 Fig — The median number of dogs within milestone 1 before rabies introduction as a function of (A) wild dog density, (B) mean lifespan, (C) mean replacement period, (D) sociability scale parameter, (E) spatial scale parameter, λ, and (F) random number generator seed, given rabies percolates (blue, milestone 1) 30 km, (green, milestone 2) 60 km, (red, milestone 3) 90 km, or (black, milestone 4) 120 km. The (magenta, all simulations) curve is the median number of dogs within milestone 1 before rabies introduction irrespective of whether rabies percolates or not. (PDF) [file pntd.0005312.s004.pdf]

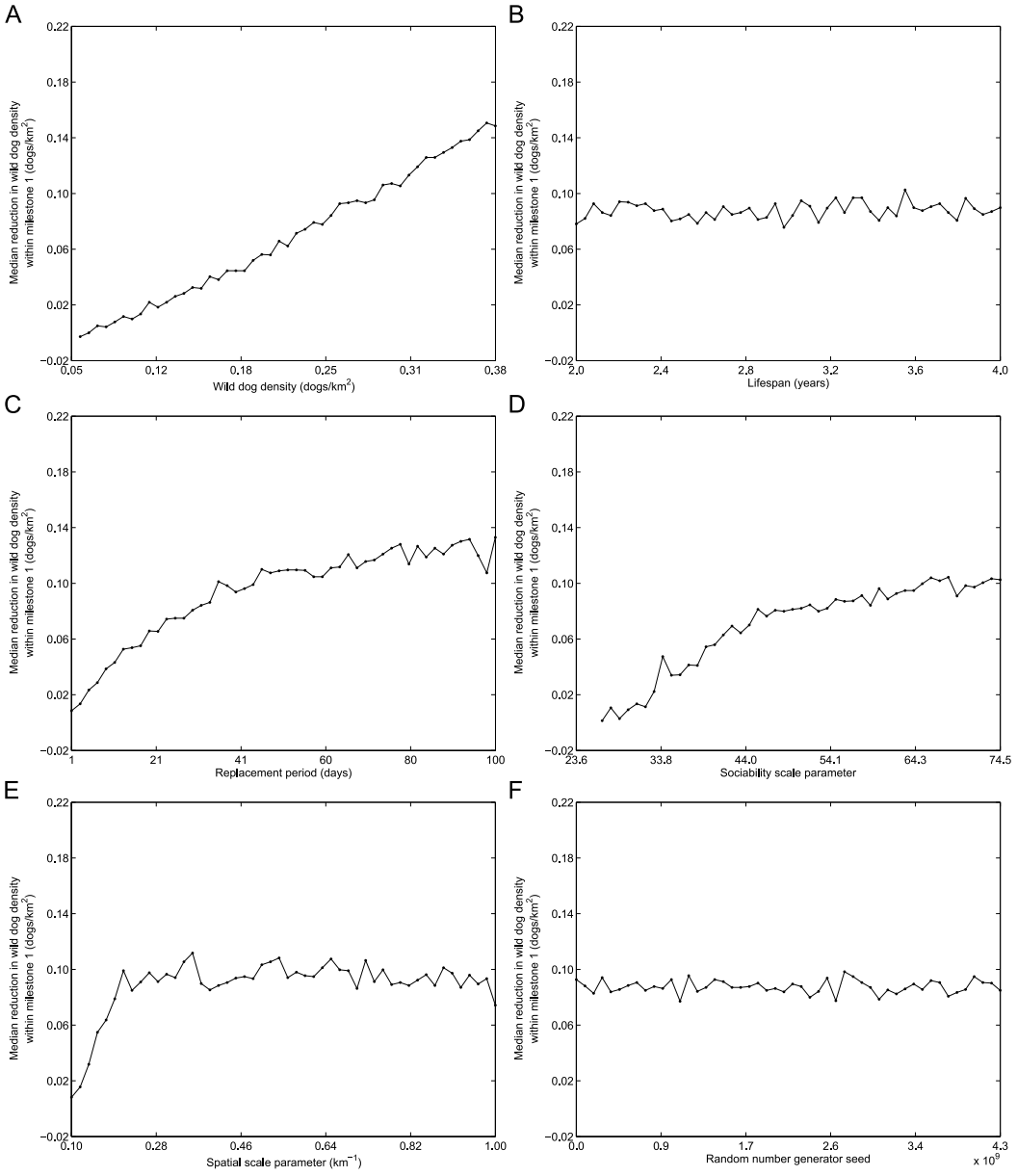

Supplement: S4 Fig — The median reduction in wild dog density within milestone 1 as a function of (A) wild dog density, (B) mean lifespan, (C) mean replacement period, (D) sociability scale parameter, (E) spatial scale parameter, λ, and (F) random number generator seed, given rabies percolates beyond milestone 4 (120 km). (PDF) [file pntd.0005312.s005.pdf]

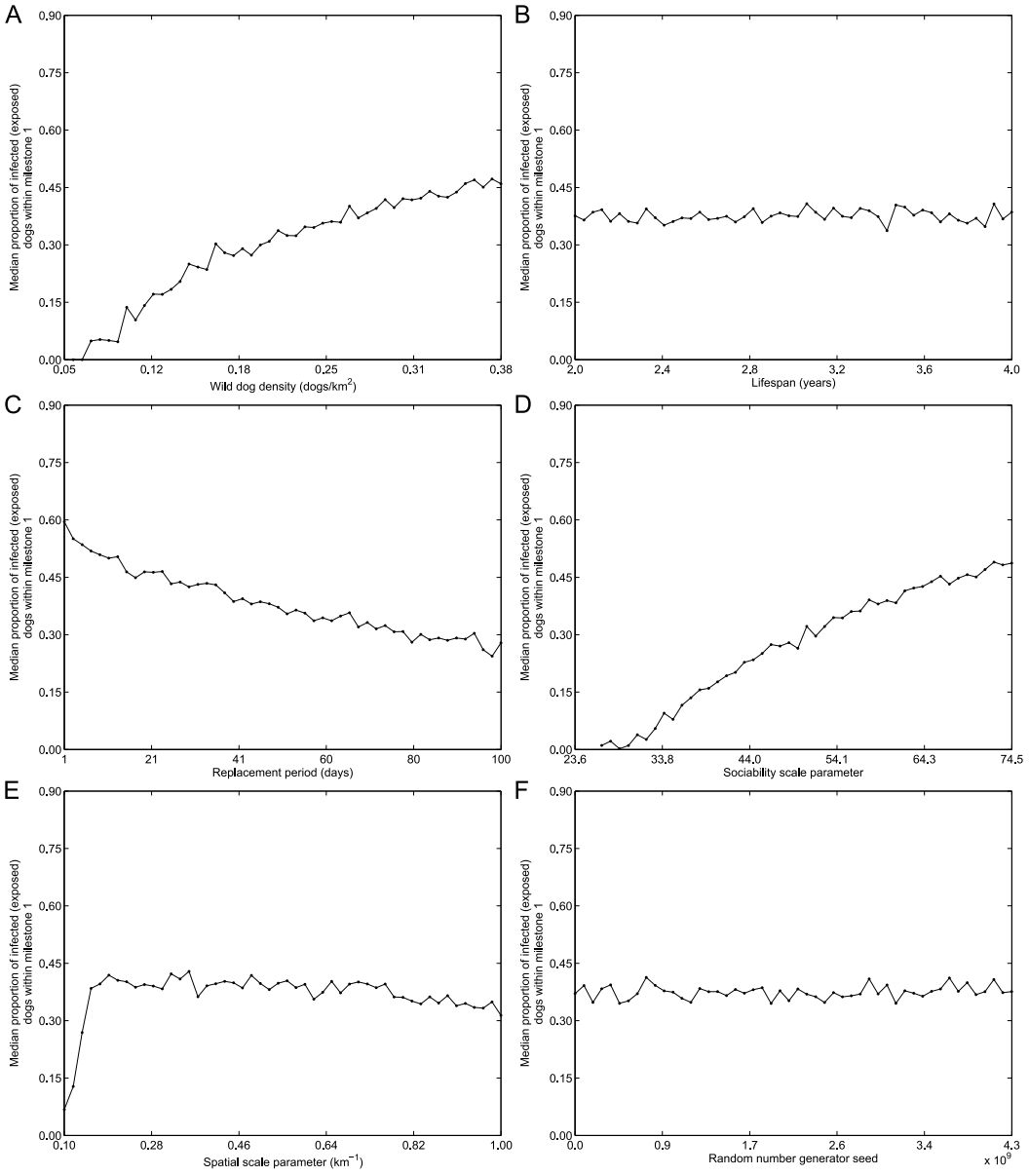

Supplement: S5 Fig — The median proportion of infected (exposed) dogs within milestone 1 as a function of (A) wild dog density, (B) mean lifespan, (C) mean replacement period, (D) sociability scale parameter, (E) spatial scale parameter, λ, and (F) random number generator seed, given rabies percolates beyond milestone 4 (120 km). (PDF) [file pntd.0005312.s006.pdf]

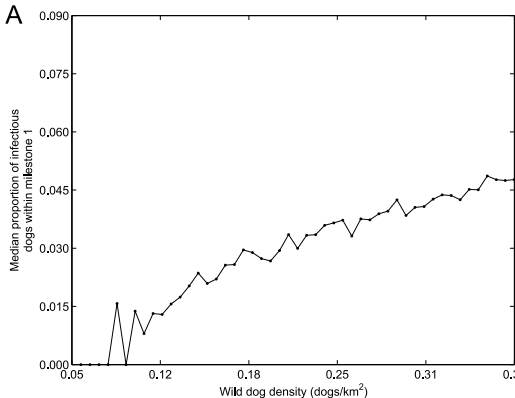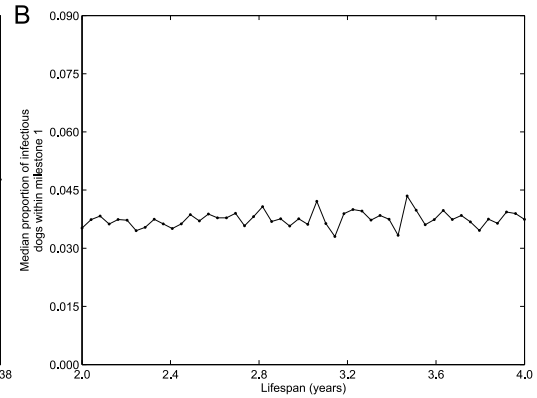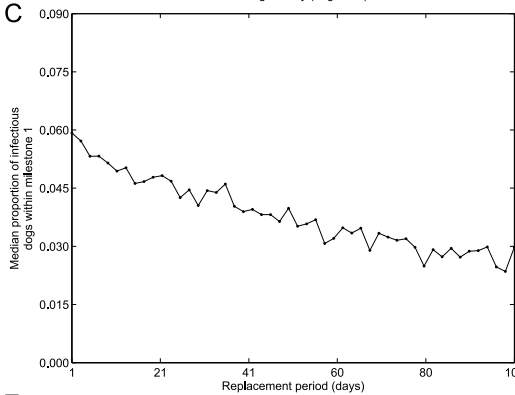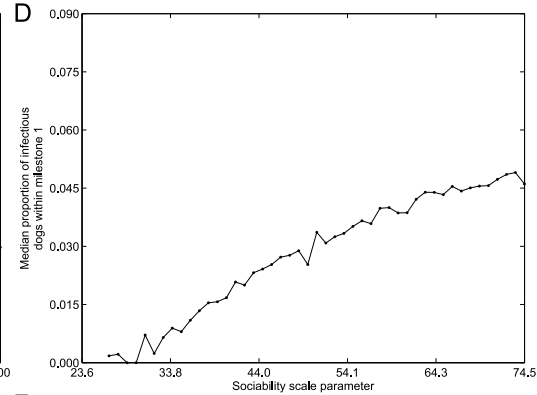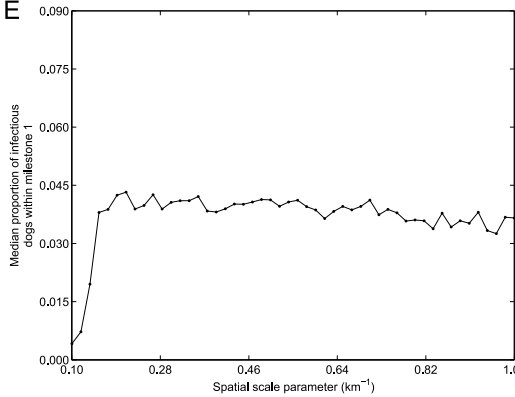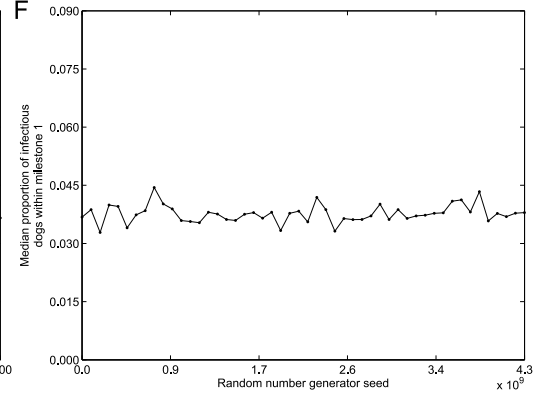

Supplement: S6 Fig — The median proportion of infectious dogs within milestone 1 as a function of (A) wild dog density, (B) mean lifespan, (C) mean replacement period, (D) sociability scale parameter, (E) spatial scale parameter, λ, and (F) random number generator seed, given rabies percolates beyond milestone 4 (120 km). (PDF) [file pntd.0005312.s007.pdf]
